# The development and evaluation of an online application to assist in the extraction of data from graphs for use in systematic reviews

**DOI:** 10.12688/wellcomeopenres.14738.3

**Published:** 2019-03-07

**Authors:** Fala Cramond, Alison O'Mara-Eves, Lee Doran-Constant, Andrew SC Rice, Malcolm Macleod, James Thomas

**Affiliations:** 1 Pain Research, Department of Surgery and Cancer, Faculty of Medicine, Imperial College London, London, UK; 2 EPPI-Centre, Department of Social Science, UCL Institute of Education, University College London, London, UK; 3 Independent Researcher, Manchester, UK; 4 Centre for Clinical Brain Sciences, University of Edinburgh, Edinburgh, UK

**Keywords:** Systematic review, automation, data extraction, graphs

## Abstract

**Background:** The extraction of data from the reports of primary studies, on which the results of systematic reviews depend, needs to be carried out accurately. To aid reliability, it is recommended that two researchers carry out data extraction independently. The extraction of statistical data from graphs in PDF files is particularly challenging, as the process is usually completely manual, and reviewers need sometimes to revert to holding a ruler against the page to read off values: an inherently time-consuming and error-prone process.

**Methods:** To mitigate some of the above problems we integrated and customised two existing JavaScript libraries to create a new web-based graphical data extraction tool to assist reviewers in extracting data from graphs. This tool aims to facilitate more accurate and timely data extraction through a user interface which can be used to extract data through mouse clicks. We carried out a non-inferiority evaluation to examine its performance in comparison with participants’ standard practice for extracting data from graphs in PDF documents.

**Results:** We found that the customised graphical data extraction tool is not inferior to users’ (N=10) prior standard practice. Our study was not designed to show superiority, but suggests that, on average, participants saved around 6 minutes per graph using the new tool, accompanied by a substantial increase in accuracy.

***Conclusions:*** **Our study suggests that the incorporation of this type of tool in online systematic review software would be beneficial in facilitating the production of accurate and timely evidence synthesis to improve decision-making.

## List of abbreviations

AUC: Area Under the Curve

CAMARADES: Collaborative Approach to Meta-Analysis and Review of Animal Data from Experimental Studies

PDF: Portable Document Format

ROC: Receiver Operating Characteristic

SyRF: Systematic Reviews Facility

## Background

Systematic review and meta-analysis are research techniques whereby as much relevant literature as can reasonably be identified on a research question is collated and analysed to give an overview of that field. In a meta-analysis, the quantitative results of the relevant research evidence are extracted from the primary research and statistically synthesised (analysed) to determine an estimate of the overall effect observed across studies and the precision associated with that effect estimate.

In order for the meta-analysis to be based on a sound dataset, the outcome data (i.e., quantitative results) need to be accurately and efficiently extracted from the primary research studies. This is often more challenging than perhaps it sounds. Studies within a review can present relevant outcome data in different ways, whether it be through providing multiple measures of the same outcome, measures at multiple timepoints, or in multiple statistical forms. These variations require skill and attention from the analyst to determine which data points need to be extracted and included in the analysis, in such a way that minimises bias and error in the selection and extraction of data. This can make the process very time consuming, even for small reviews; the labour required is obviously compounded in very large reviews, such as those seen in preclinical research.

A further complication is the actual presentation of the data, as different studies will report the outcomes in different ways, such as graphical plots, in tables, or as text. Whilst it might be difficult to aid reviewers in terms of *selecting* which pieces of data to extract through a software program, as this will inevitably vary from review to review, we considered there to be clear potential to improve both the speed and accuracy of extraction of outcome data from the included studies once the required outcomes have been identified. This report is of an evaluation of a tool designed to assist specifically with the extraction of outcome data from graphical plots, as these can be particularly time-consuming and prone to error ^1, 2^. We acknowledge that there is a variety of different ways that papers are presented (online, paper only, etc.), but we focused this work on the challenge of extracting data from papers published using the PDF file format. This format is the most ubiquitous electronic publishing medium for papers and we therefore see greatest efficiencies from improving software support in this area.

### Motivation for this work

The use of systematic reviews is commonplace in clinical research, for example through Cochrane, where they are seen as the pinnacle of high-quality research synthesis, and are used frequently in clinical decision making ^3^. In preclinical and *in vivo* fields, however, systematic reviews are less prevalent, but arguably can be just as useful, for example by guiding future research and bridging the gap between the quantity of research produced and the amount that can be effectively used by an investigator. Whilst there are some research groups pioneering the use of systematic review in preclinical fields (e.g. CAMARADES) systematic reviews have not yet gained the widespread acceptance that they have in clinical research ^4^.

One of the key criticisms of systematic reviews is that, once published, they can quickly go out of date ^5^. Whilst this is true for clinical systematic reviews, it is especially true for preclinical reviews due to the sheer volume and accrual rate of preclinical literature, which means that a preclinical systematic review and meta-analysis is likely to take a longer time to complete than a clinical one. For example, in a recently completed systematic review of neuropathic pain, data from 229 clinical trials required extraction ^6^, whereas for the corresponding on-going preclinical systematic review data are being extracted from approximately 6000 studies. Therefore, to improve the feasibility, acceptance and usefulness of systematic reviews, methods and technologies need to be developed to speed up the process, and these advances need to be made without damaging the quality of the resultant review, and be easy and simple to disseminate on a wide scale.

Once the studies for inclusion in a systematic review have been identified, the process of ‘data extraction’ (or ‘data collection’) begins ^7^. This usually involves the abstraction of data from each included study in a systematic and standardised way, from the published reports of the studies, into software from which the data can be analysed as a whole. As the synthesis of findings is conducted using these extracted data, it is vital that the data are extracted reliably. To aid reliability, data are usually extracted by two people working independently, and checked against one another. There is empirical evidence that mistakes made at this stage of the review process can affect effect estimates, and hence, review conclusions ^2^.

Outcome data can be quite challenging to extract. Transcription errors are a common problem, with some errors not being detected until after the systematic review has been published ^2^. Moreover, some outcome data are only reported in graphs, and systematic reviewers must therefore measure values from the graphs as accurately as they can and record the results. The time taken is an important component of the cost of conducting systematic reviews and reduces their currency. While most results from clinical trials tend to be reported in tabular form, some diagnostic test accuracy studies only report some aspects of their results in graphical form; and in the preclinical field, the reporting of results in graphs alone is commonplace.

The use of bespoke online software for conducting systematic reviews is becoming increasingly standard practice, with tools such as Covidence, DistillerSR, EPPI-Reviewer and SyRF offering support for a range of review types in commonly available browsers. None of these platforms support the extraction of data in graphical form, however, and reviewers need therefore to use other tools to extract data from graphs, and then transcribe this information into whichever tool they are using for synthesis. This causes two problems. First, additional effort is needed on the part of reviewers to determine which application to use, to use it consistently across the reviewing team, and then to copy data back for synthesis. Second, there are no graphical extraction tools especially written for the types of data and workflows encountered in systematic reviews. The data can therefore require some transformation before being suitable for incorporation in the review. Given these challenges, and the growing infrastructure of browser-based systematic review applications, we decided to evaluate the possibility of utilising browser technologies to improve the efficiency of data extraction from graphs. To do this, we: 1) identified relevant technologies; 2) compiled a dataset for evaluation; 3) developed a pilot user interface; and 4) undertook an evaluation of the user interface in terms of its efficiency and accuracy as compared with other extraction methods. The aim was not to create a new tool that was ready for widespread deployment, but to inform future development decisions, based on the evaluation, as to the utility of integrating such a tool in systematic review software.

## Methods

### Research aim

To determine if machine assistance for extracting data from a variety of graph types using a tool that has been developed and customised for systematic review needs has a meaningful impact on a) the time taken and b) the accuracy of the data extracted, compared to current methods (typically using desktop measuring software).

As there are no tools for graphical data extraction that have been developed specifically for systematic reviewers to use, we developed a pilot tool for evaluation purposes that is described below. Our primary interest, however, is in the relative performance of a tool which is designed specifically for data extraction in the context of systematic reviews (rather than this specific tool *per se*).

### Research questions

1. Through a comparison of the use of our customised tool with the user’s current method of outcome data extraction, is there a notable difference between the two methods in the following metrics?
  a) Time taken
  b) Accuracy
2. Do participants in the evaluation (with a pre-existing interest in and knowledge of systematic review) consider this to be a viable or preferential approach compared with their current practice?

### Participants and recruitment

We attempted to recruit participants from collaborators, colleagues and students on Masters-level systematic review modules using direct communication, email, social media and face-to-face interactions at conferences. The recruitment strategy targeted people who were known to have training and/or experience in conducting quantitative systematic reviews. No formal sample size calculation was performed because in this study the variation between individuals in the time taken in data extraction was not previously known, but we reasoned that a minimum of 10 participants assessing each of 23 graphs using 2 different approaches would give insights to the strengths and weaknesses of each approach, and of areas for future development.

We provided participants with an information sheet ( Supplementary File 2) and consent form ( Supplementary File 3), which had to be signed and returned before participation could commence. As complete datasets were most useful, participants were encouraged to complete the trial in its entirety.

### Identifying graph types

To guide the development of the tool we first established the structures of graph and data typically featured in research papers, starting with the preclinical literature, where we consider the challenge of extracting data from graphs to be particularly acute. To do this we selected 34 papers ( Supplementary file 6) identified in the context of systematic reviews in two different preclinical fields (animal models of neuropathic pain and animal models of D-galactose-induced aging). Papers were selected covering a range of dates to account for any changing publication patterns within the literature. These were hand-checked by F.C. to ensure that they would be relevant for our purpose (i.e. an original research paper that could be included in a review and contained outcome data presented in graphs). The number of papers required at this stage was not predetermined; instead, we continued collecting graph types until no new graph had been found for 10 consecutive papers.

Two team members that do not work in preclinical research (A.O.E. and J.T.) checked the types of graphs collated to determine whether the range of graphs in their disciplines (clinical and public health research – Supplementary file 7) were represented. The team identified that area under the curve (receiver-operator curve) plots, which are common in diagnostic test accuracy systematic reviews, were not represented, and these were added to the list of graph types.

### Developing the web-based tool for graphical data extraction

We developed requirements for the graphical data extraction tool and chose a browser-based solution for ease of deployment during evaluation and because, should that evaluation prove positive, the code could be integrated within web-based systematic review software such as those mentioned above. The two main requirements were that: a) the user interface should display PDF files and support the selection of graphs from which data would be extracted; and b) the user should be able to extract data from the graphs by specifying axes values and data types, and then by clicking appropriate points on the screen with a mouse. In terms of browser requirements, we decided that we would require HTML5 compliance, since most platforms now support this standard, and if we needed to support older browsers the cost of development would have been prohibitive.

We developed the graphical data extraction application using two existing JavaScript libraries: PDF.JS (version 1.5.188) and WebPlotDigitizer (version 3.8 ^8^). PDF.JS is a widely used library for displaying PDF files in web browsers. We used this library to display the graphs to evaluation participants and to allow them to draw a box around selected graphs. WebPlotDigitizer is a program that can extract data from graphs that are uploaded in a PNG or JPEG format, so we used JavaScript to ‘send’ the graph image to WebPlotDigitizer, and this library was customised to support our workflow and the data types common in systematic reviews. We now describe in more detail the workflow in the customised tool, and the software development undertaken to support it.

Our starting point for the design of this workflow was that users would be extracting data from a PDF file and, as part of this process, would encounter graphs within the file from which they would need to extract numeric data. We simulated this initial point of entry by using PDF.JS to display PDF files. This tool can display the majority of PDF files, and we encountered no problems when using it. We modified the default display of the tool in two ways for the purposes of this evaluation. First, we supressed the appearance of buttons and functionality that were unnecessary for our purposes (e.g. the ability to ‘zoom’ into / out of the page). Second, we wrote functionality that enabled users to draw a box around content in the PDF with a mouse – in our use scenario, users were expected to draw a box around a graph – and extract this part of the PDF file as an image to be used in the next part of the workflow. Figure 2 shows the display of graphs in the PDF.JS tool with a ‘box’ drawn around the graphic. The user then clicks the box to move to the next part of the workflow where we incorporated the WebPlotDigitizer tool ^8^. WebPlotDigitizer is an online tool which supports the extraction of numeric data from many types of graphs. We customised the tool so that it supported both the data types that systematic reviewers use explicitly, and also our expected workflow.

After drawing a box around their selected graph and clicking the graph, the user moves into the user interface of WebPlotDigitizer ^8^. Our modifications to WebPlotDigitizer fell into three main areas which are outlined in detail below: 1) user selection of specific data types and structures at the outset; 2) the addition of an interactive data table, which captures the data in a structure which is suitable for use in subsequent meta-analyses; and 3) data export.

We modified the normal point of entry to WebPlotDigitizer to display a menu of data types, series and data points for users to specify exactly what type of graph they were going to extract data from ( Figure 3). In the example screenshot in Figure 3, we can see that the graph has three data points for each of four series, and each data point has a mean and confidence intervals around it. The user specifies this information, as well as whether the graph has one or two axes (in the example, there is only one axis: the y-axis). The tool then utilises the standard functionality of WebPlotDigitizer which supports the calibration of axes. This involves clicking the mouse on the origin (i.e. the bottom left hand side of the graph in the example) and the highest value in the axis (in the example, on the 10). The user then enters the values and the software can calculate the correct values for the positions of any mouse-clicks in between the two.

After specifying the position of the axis (or axes) and calibrating them, the user then enters the main data extraction screen ( Figure 4). Here, our development work focused on a new interactive data table which can be dragged to any part of the screen, or docked on the far right-hand-side ( Figure 5). The structure of the data table matches the parameters entered previously; in the example, we have three columns for data: the mean, and the upper, and lower confidence intervals. This data structure is multiplied by the number of series and the number of data points that have been specified by the user. The titles of the series are editable text boxes and the user can use the tab key to move between them, entering series titles.

The user then clicks in the cell that they want to enter data into and can then use the mouse to click data points on the graph. The user interface automatically advances between cells. For example, in the data table shown in Figure 5, the user would click in the top cell in the ‘mean’ column and click on the first data point for ‘Model + vehicle’. This value then appears in the relevant cell. The ‘active’ cell automatically advances to the upper confidence interval, which is filled in when they click on the relevant point on the graph. Note that the data table is ‘aware’ of the various data types that it contains and calculates this value as a difference between the mean and the corresponding value on the y-axis, rather than just the y-axis value. The data table can have radically different structures; for example, it can capture both individual and aggregate level data when necessary ( Figure 6).

The ‘point-and-click’ interface, working alongside the data table means that the user is able to extract the relevant numeric data from a graph in a matter of seconds. Moreover, in order to assist with accurate mouse positioning, WebPlotDigitizer ^8^ contains by default a window on the top right which shows a ‘zoomed in’ display of the current mouse position.

The final piece of development that we undertook was to save the data back up to the server ready for analysis.

Development took place iteratively during November 2016 to June 2017 by a highly experienced JavaScript developer (LD-C) resulting in the customised tool which was placed online for evaluation in June / July 2017. The integration and customisation of the two existing JavaScript libraries, PDF.JS (version 1.5.188) and WebPlotDigitizer (version 3.8 ^8^), resulted in a prototype workflow designed specifically around the needs of systematic reviewers. The aim was not to create a new tool that was ready for widespread deployment, but to inform future development decisions, based on the evaluation, as to the utility of integrating such a tool in systematic review software.

### Evaluation design

We used a non-inferiority trial design to evaluate the graphical data extraction application, with each participant extracting data from graphs using their current methods of data extraction and the new, customised graphical data extraction application. The study was approved by the UCL Institute of Education Research Ethics Board (reference REC 944.)

Our primary aims were to determine whether there were differences in time taken and accuracy between a user’s current approach and the new approach to data extraction. We also sought feedback from users as to the usefulness of the new, customised tool.

We identified 5 broad classes of graph and created 23 examples (5 bar, 5 line, 5 scatter, 3 dot plot, and 5 box and whisker) ( Supplementary File 5) in SigmaPlot version 10 using fictitious data and expressed to 3 significant figures, so that the true value for each data point was known; the ‘new tool’ condition had an additional class of graph, ROC/AUC, for which 4 graphs were created. Participants were required to extract data from graphs using both their current methods of data extraction and the new graphical data extraction application. For each method of extraction they worked though all 23 graphs in the same order (plus the 4 AUC/ROC graphs in the ‘new tool’ condition, and whether they started with the new method or their current method (defined as their preferred method that is used most often when extracting data) was determined at random by software code embedded within the study website.

### Current methods condition

**Figure 1.**
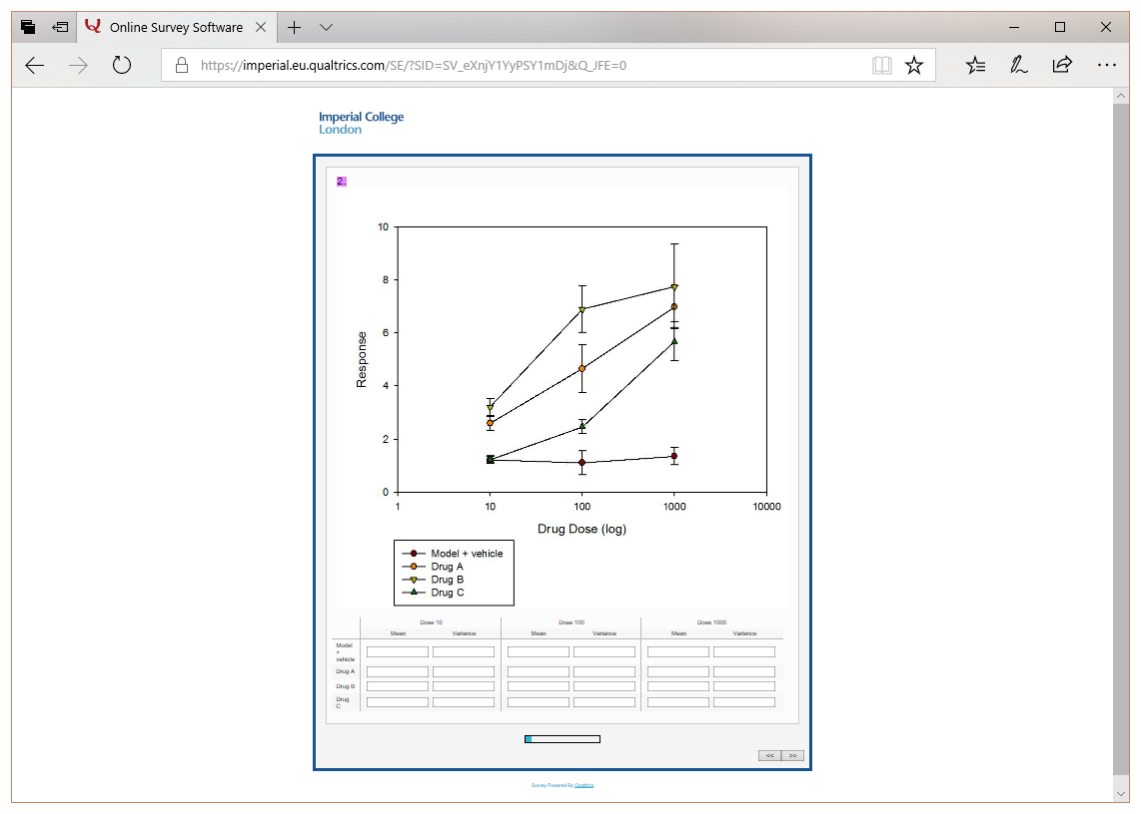
The ‘current methods’ data collection tool in the Qualtrics platform.

The evaluation aimed to compare the purpose-built workflow, described above, with current practice. Because ‘current practice’ varies from person to person, we did not specify exactly which method participants should use, as we wanted them to use the one that they would naturally use – whether that was a specific tool, or simply holding a ruler against the computer monitor. Participants were therefore instructed to extract data from plots in this condition using whatever methods they typically currently use. Participants reported that the following tools were used in this condition: Universal Desktop Ruler (3), In-built Adobe Acrobat measuring tool (3), WebPlotDigitizer (2), and Grabit (1). The Qualtrics survey platform was used to collect data for this condition, whereby the graphs were uploaded alongside a table where the participant was asked to record their extractions. This software allows an accurate timing per graph to be collected. Figure 1 is a screenshot of one of the graphs with the table for entry of extracted data shown below. A copy of the platform as presented to participants can be found at https://imperial.eu.qualtrics.com/jfe/form/SV_eXnjY1YyPSY1mDj.

**Figure 2.**
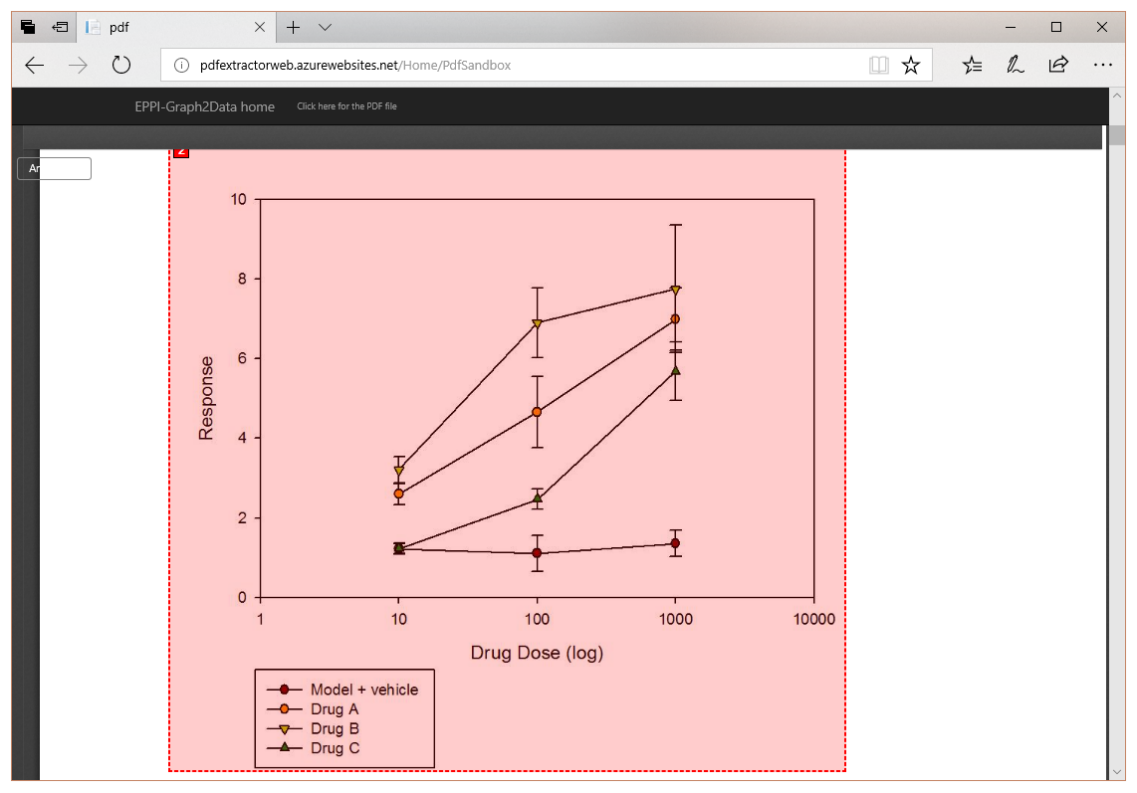
The graph as displayed in PDF.JS.

**Figure 3.**
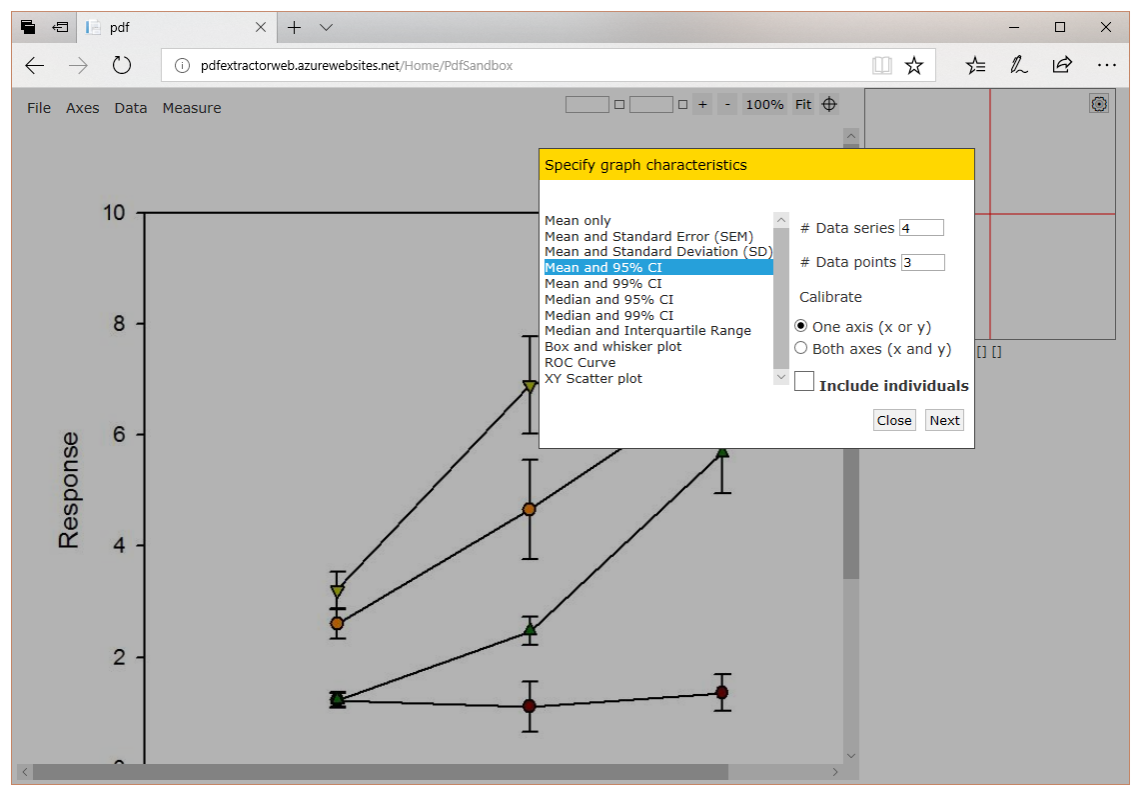
Specification of graph characteristics.

**Figure 4.**
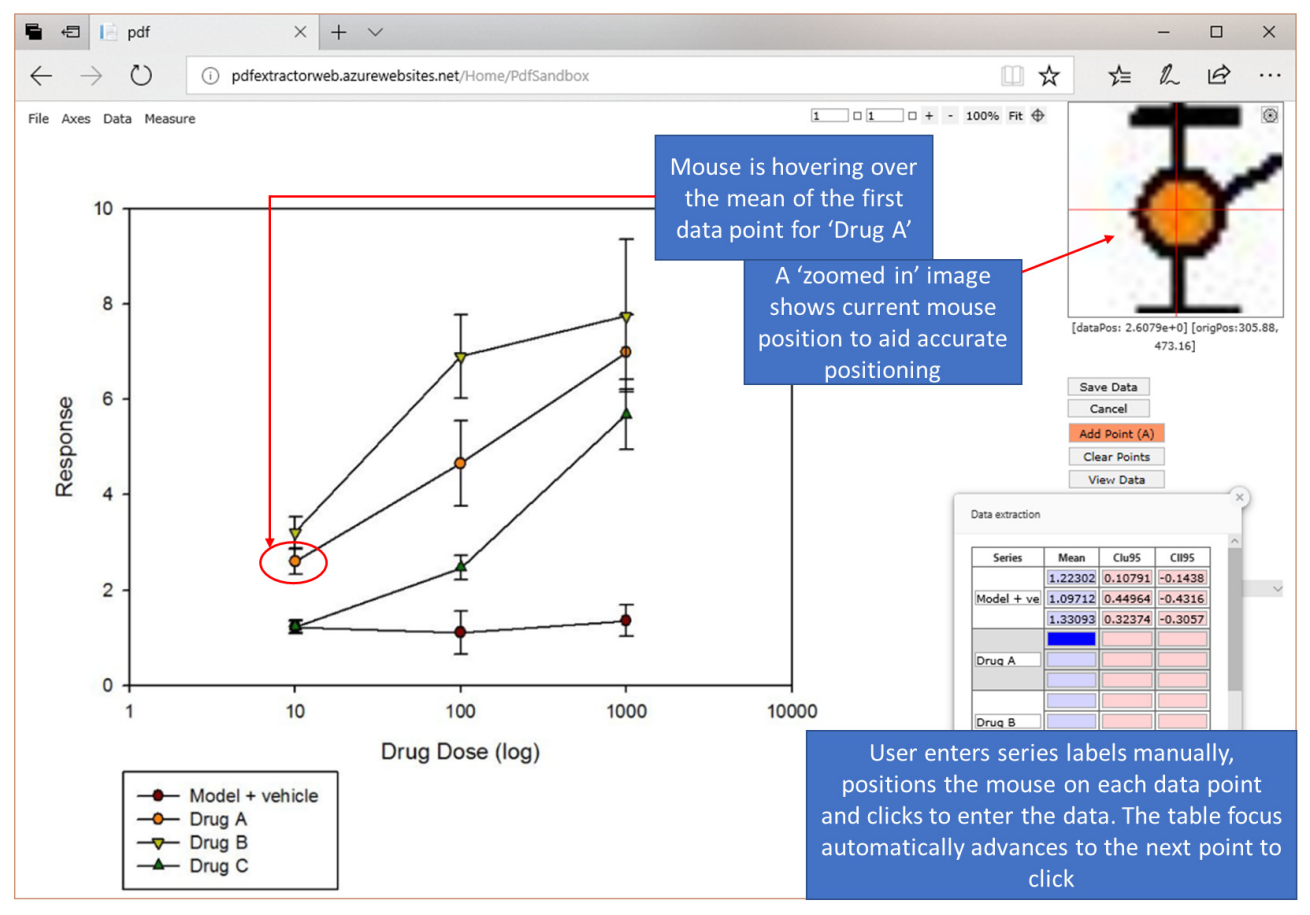
Data extraction.

**Figure 5.**
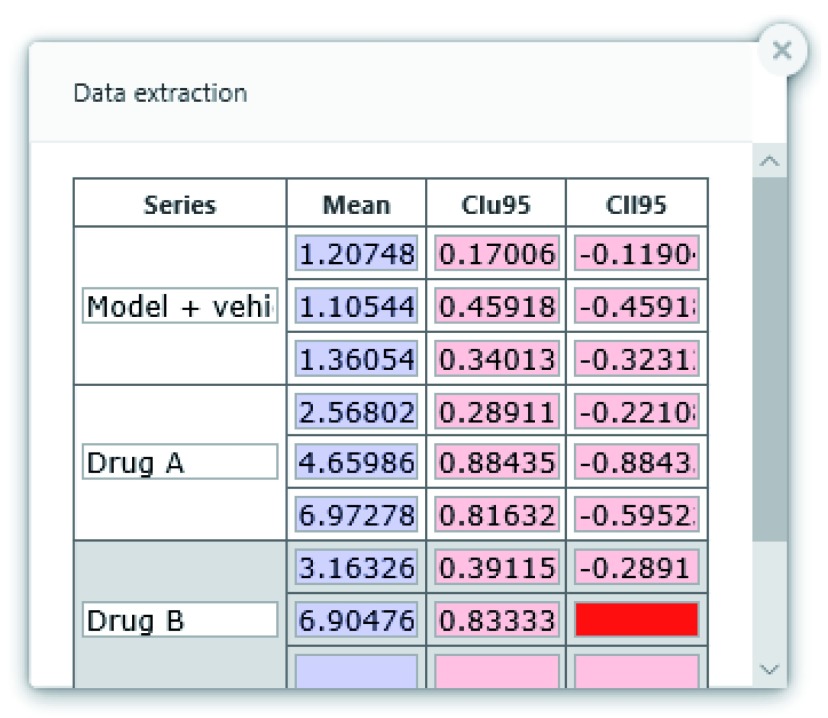
Data table.

**Figure 6.**
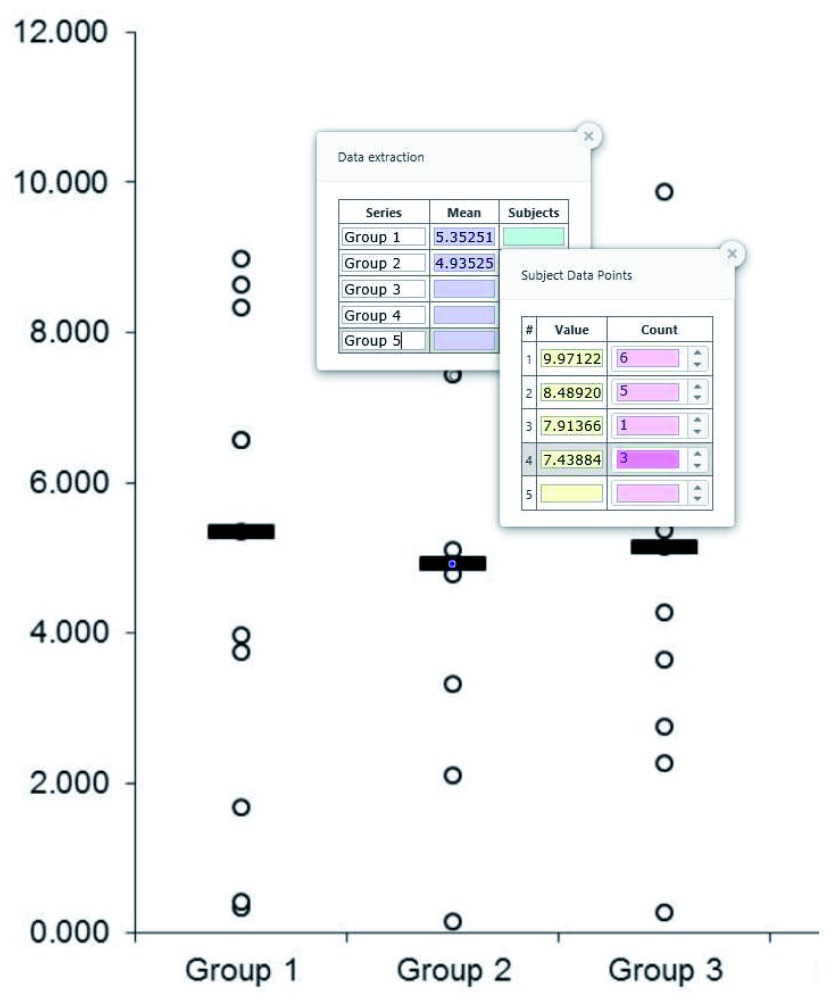
An alternative type of data table, supporting individual and aggregate level data.

Because of the challenges in manual data extraction from ROC/AUC graphs, these were not offered in this set.

### New graphical data extraction application condition

The graphs for the new graphical data extraction application were the same and in the same order as the current methods condition, with the addition of 4 AUC/ROC graphs.

The evaluation website for the graphical data extraction application was hosted at: http://pdfextractorweb.azurewebsites.net/. As well as supporting the data extraction problem itself, the graphical data extraction application measured the time that participants spent extracting data from each graph automatically. Participants were given comprehensive instructions, including a YouTube tutorial video ( https://www.youtube.com/watch?v=tzg-NUV-wcg&feature=em-upload_owner) and instructed not to leave the platform running when not in use, as this would affect the accuracy of the time measurements. Data validity were also part of the subsequent analysis

### Participant experience

We used a qualitative survey hosted on the surveymonkey.com platform. The questions focused on the background experience of the participants and their perceptions on the ease, speed, and features of the tool. Participants were also asked to indicate their preferred method for future extractions and were able to submit suggestions for development of the tool. This was filled in after completion of the trial. The questions on the survey are presented in Supplementary File 1: https://www.surveymonkey.co.uk/r/G5XYQDS.

### Quantitative analysis

We used a non-inferiority trial design to seek to demonstrate that the novel process (the use of a graphical data extraction application) was not meaningfully worse than the existing process (current methods of data extraction). Data were analysed in Microsoft Excel. The analysis process is outlined below.

To establish the time taken to extract the data for each method we used a within-subjects design. As participants were required to extract the same data from the same graphs in each condition, it was possible to directly compare how long it took using each method of extraction. To measure differences between approaches we calculated by subtraction, for each graph and for each participant, the difference in time taken between each approach, such that a positive value would indicate that the current methods took longer than the new method. Then, for each graph we calculated a mean difference in time taken across participants, along with the standard deviation; and we also calculated the total time taken for all 23 graphs represented in both conditions, and expressed this as minutes.

Note that analysis of the difference in time taken for the two conditions could not be computed for the four AUC graphs because they were only presented in the new graphical data extraction application condition (i.e., we do not have data for the four AUC graphs in the current methods condition).

To establish the accuracy of data extraction, we compared extracted values with the known true values used to render the graphs.

We first defined the tolerable bounds of an ‘accurate’ extraction for each graph ( Supplementary File 4). We calculated the bounds as 1/20 ^th^ of one increment in the scale of the graph outcome axis (usually the y-axis). For example, if the outcome axis scale had increments of 10, then a bound of ± 0.5 around the true data point was set. If a given true data point had the value of 6, with a tolerable bound of ± 0.5, then we would accept any value between 5.5 and 6.5 as accurate for that data point. The bounds for each graph are shown in Supplementary File 4. Extracted data points lying on or within these bounds were considered accurate, while those above the upper bound or below the lower bound were considered inaccurate.

In a real systematic review, data extraction is usually performed by two individuals working independently because 100% accuracy in data extraction cannot be guaranteed; errors of one extractor can be detected when disagreement is observed with the other extractor, and these data points identified for third person reconciliation. For each data point, we determined whether 80% or more participant responses were within the tolerable bound. For each graph, we were then able to determine what proportion of data points were ascertained with sufficient accuracy.

To give a summary estimate of differences in the accuracy of data extraction using the different methods, we calculated the difference between the percentage of accurate data points using the new method and that using conventional methods. We determined in advance that we would consider that the new method was inferior to current methods if the point estimate of sufficient accuracy was greater than or equal to 5% lower than current methods (i.e., the new, customisedtool would be considered inferior if SufficientCurrentMethod – SufficientNewMethod ≥ 5%). Under such circumstances, substantial redesign of our approach would be required.

We also calculated an odds ratio for obtaining a sufficiently accurate data point in the new method compared to the current method as: (SufficientNewMethod/ InsufficientNewMethod) / (SufficientCurrentMethod/ InsufficientcurrentMethod), where the values represent the number of data points that were of sufficient (or insufficient) accuracy in the two conditions (new and current methods).

### Qualitative analysis plan

A secondary aim of the project was to consider users’ reactions to the new, customised tool. Analysis of the multiple-choice questions involved examination of frequencies and percentages of participant responses. Analysis of the open-ended text responses involved coding the text into categories (themes) that were derived from the data (i.e., not *a priori*); for example, free text comments about how quickly the participant extracted data were coded as relating to the theme of ‘speed’. The frequencies of themes mentioned across participants were examined. To protect the anonymity of the participants and encourage completion, the survey data were not linked to the responses from the data extraction conditions.

## Results

### Recruitment

Emails were directly sent by a members of the research team to more than 50 people. We are unable to state how many people were exposed to the social media adverts, and therefore cannot provide an accurate number of how many people were indirectly approached.

A total of 32 consent forms were returned. Of these individuals, 10 completed the trial, 9 never started the trial, 7 partially completed the trial and 6 were excluded or dropped out. Recruitment commenced 30/06/17 and was completed 01/10/17. Data for a total of 10 participants were included in the analyses. The relatively high drop-out rate can be explained by the time demands of the evaluation. The evaluation – and especially the ‘current methods’ component took some participants several hours to complete. This was necessary in order to collect sufficient data to be able to compare the two approaches across so many different types of graph, but it did affect recruitment and retention.

### Time

As described in the methods, we calculated the difference in times as the time for the current methods condition minus the time for new graphical data extraction tool within a participant, so that a positive value would indicate that the current methods took longer than the new method. The mean of these differences across participants was calculated to give $\left(\bar{X_{g}}\right)$ (in seconds); the results of which are reported for each graph in Table 1.

**Table 1.** Mean and standard deviation of the time difference for each graph across n participants.

Graph number	Mean $\left(\bar{X_{g}}\right)$ time difference, s	Standard deviation	Participants, n
1	180.02	240.55	10
2	363.60	255.27	10
3	314.59	225.16	9
4	140.22	121.24	10
5	113.68	132.87	10
6	486.54	298.17	10
7	463.91	410.30	10
8	167.77	153.72	9
9	332.57	252.34	8
10	546.28	649.84	7
11	412.55	243.81	9
12	564.25	820.52	8
13	210.15	169.28	8
14	377.24	466.44	8
15	281.76	331.66	8
16	478.74	404.05	8
17	691.20	738.84	7
18	119.62	104.42	8
19	93.34	151.23	9
20	650.31	750.77	9
21	469.19	804.92	8
22	373.28	462.56	9
23	270.24	258.40	8

Note: A positive time difference indicates that the current methods condition took longer than the new graphical data extraction application method condition.

For each graph, the average time taken was less when using the new graphical data extraction tool compared with the usual approach used by participants, with some differences of more than 10 minutes. Overall, the mean time taken to extract data was 352 s (5 min 52 s) less using the new, customised tool than using the conventional approach (median, 364 s; IQR, 180–469 s; range, 93–691 s).

### Accuracy

As described in the Methods, we considered whether a given data point was sufficiently accurate if at least 80% of participants’ responses fell within a tolerable boundary around the true value. The number of data points that were of sufficient accuracy or insufficient accuracy were summed for each graph. The results for each graph, presented by condition, are shown in Table 2. Recall that the new tool would be considered inferior if SufficientCurrentMethod – SufficientNewMethod ≥ 5%. Overall, the current method ascertained data with sufficient accuracy for 41% of data points, compared with 70% for the new approach, for a difference of -29%, which is substantially better than our prespecified non-inferiority value of 5%. (Here, anything less than 5% difference is favourable to the new method). The odds ratio of getting a sufficiently accurate data point compared to an insufficient data point in the new method compared to current methods was 3.34 (95%CI = 2.51, 4.44).

**Table 2.** Frequency per graph of data points deemed sufficient accuracy or insufficient accuracy, with percentage of data points that are sufficient accuracy, by condition.

Graph	Current methods condition			New graphical data extraction application condition		
	Sufficient accuracy	Insufficient accuracy	Percent sufficient data points	Sufficient accuracy	Insufficient accuracy	Percent sufficient data points
1	3	1	75.00%	4	0	100.00%
2	16	8	66.67%	18	2	90.00%
3	7	5	58.33%	7	5	58.33%
4	1	9	10.00%	10	0	100.00%
5	8	4	66.67%	6	0	100.00%
6	15	33	31.25%	17	0	100.00%
7	14	6	70.00%	5	15	25.00%
8	9	11	45.00%	9	11	45.00%
9	16	16	50.00%	18	0	100.00%
10	could not match data so removed from analysis					
11	0	30	0.00%	0	20	0.00%
12	3	33	8.33%	20	14	58.82%
13	10	10	50.00%	20	0	100.00%
14	37	3	92.50%	14	0	100.00%
15	could not match data so removed from analysis					
16	12	28	30.00%	10	12	45.45%
17	22	33	40.00%	8	12	40.00%
18	0	12	0.00%	5	5	50.00%
19	10	2	83.33%	12	0	100.00%
20	could not match data so removed from analysis					
21	22	38	36.67%	27	9	75.00%
22	12	30	28.57%	14	0	100.00%
23	10	14	41.67%	20	0	100.00%
**Totals**	**227**	**326**	**41.05% ^a^**	**244**	**105**	**69.91% ^a^**

Notes: Three of the graphs (10, 15, 20) had incompatible data because participants in the new graphical data extraction application condition selected too many different data input types, so a comparison could not be made. The total number of data points in the two conditions differs due to issues including missing data or incorrect selection of graph type in the new graphical data extraction application condition. ^**a**^This value represents the mean for this column, not the total.

### Survey results

A total of nine participants completed the qualitative survey. They were employed at a higher education institute (n=3), by a governmental agency (n=2) or were students (n=3 doctoral and n=1 masters). Their disciplines were preclinical science (n=4), statistics (n=1), clinical science/medicine (n=2) and social sciences (n=2). All had performed at least one stage of a systematic review and seven stated they had extracted outcome data previously. Tools previously used for extracting data from graphs included the universal desktop ruler (n=3 participants), Adobe measuring tool (n=4 participants), Web Plot Digitizer (n=3 participants) and Excel Grabit (n=1 participant). Three participants stated they had not previously extracted graphical data. Unfortunately, because the survey and trial data were not linked, we could not explore whether the background or experiences of the participants’ might have been associated with their performance in the trial.

**Figure 7.**
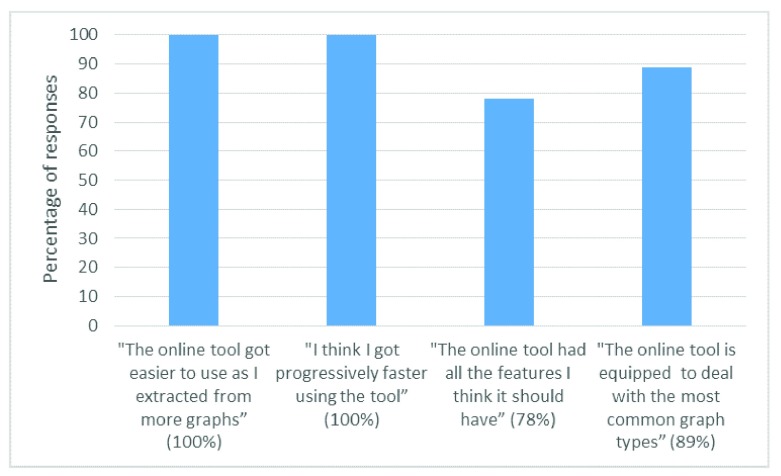
Satisfaction with the features of the new graphical data extraction tool: percentage of respondents who ‘agreed’ or ‘strongly agreed’.

The percentage of respondents that either ‘agreed’ or ‘strongly agreed’ with statements evaluating their satisfaction with the features of the new graphical data extraction tool are depicted in Figure 7. They show strong support for the tool as compared with other methods, although these and subsequent answers suggest that additional development may be needed. Raw data is available on Zenodo ^9^.

All respondents indicated that if they had to extract a third set of similar graphs using just one of the methods they would choose the new online tool. In a free text box they were asked why this selection was made. Comments referred to speed (n=7), accuracy (n=4), and ease of use (n=5).

Lastly, participants had an option to submit suggestions for improvement of the tool; these included bug-fixing, an undo button, functionality of plotting the points, and an interface to allow the tool to interact with a data storage tool.

## Discussion

### Summary of findings

We have shown that our new graphical data extraction tool ^10^ is not inferior to users’ preferred current approaches. Our study was not designed to show superiority, but suggests that, on average, participants saved around 6 minutes per graph using the new tool, accompanied by a substantial increase in accuracy. Indeed, that gain in accuracy is likely to be accompanied by further time-saving, as the number of outcome measures identified for reconciliation by a third reviewer will fall as a consequence. If our findings are confirmed, this would have profound implications for the conduct of systematic reviews where extraction of data from graphs is required. Our tool also received positive feedback from users in terms of its ease of use, fitness for purpose and perceived efficiency.

### Evidence of feasibility of further development and dissemination

For a new technology to be worth developing and disseminating, at least two conditions need to be in place. Firstly, the technology must be not inferior to existing tools. Secondly, the technology must be seen by the end users as preferable to existing tools. We believe that this study provides sufficient evidence that these two conditions have been met.

The potential cost- and time-saving aspects of the graphical data extraction tool are likely to be substantial. The results showed a mean reduction of nearly 6 minutes in time taken to extract data from graphs compared to existing methods, which could translate to a substantial time saving per systematic review publication, due to reduced reviewer time. In practice, this time saving would be amplified, as it is advised that data in systematic reviews should be extracted by a minimum of two reviewers to reduce errors ^1^ and potentially even a third reviewer to resolve discrepancies.

Furthermore, as the graphical data extraction tool showed a considerable improvement in accuracy; this will also decrease time as the third reviewer will have fewer discrepancies to resolve.

Aside from the time-saving aspect, the improvement in accuracy alone is compelling evidence for the further development of the software, as it ultimately may lead to more precise systematic reviews and meta-analyses. In line with Jelicic Kadic *et al.*, who evaluated an electronic data extraction tool with a paper-based method, we found that the use of a graph extraction tool leads to more accurate data extraction ^11^.

We note that the few graphs for which graphical data extraction application had very poor performance were cases in which some participants had selected completely the wrong graph type; this means that our estimates for the accuracy of data extraction from graphs for the new graphical data extraction application condition are considerably below that which is probably likely in real life conditions. It also suggests that some training or further guidance on graph type selection within the tool (as depicted in Figure 3) is required.

Lastly, the qualitative survey provides evidence that reviewers prefer the new data extraction tool, described hereby, to several desktop electronic methods of data extraction that are currently in use. This suggests that the tool will be acceptable and credible to the proposed users, which is necessary for its uptake.

Ultimately, there is strong evidence from the trial of the graphical data extraction tool that the further development and dissemination of this technology is worthwhile. The initial costs of implementation, training, and monitoring, would be offset by the impact of widespread use, leading to increased output of accurate systematic reviews, especially in preclinical topics where a large proportion of the outcome data are extracted from graphs.

### Future work

As it currently stands, the technology developed here has limited ‘real life’ use. For it to become a useful part of the systematic review process it would need integration with other platforms used to facilitate systematic review and meta-analysis. An example is the SyRF platform (CAMARADES) which allows for screening and annotations for risk of bias using technology developed in other work packages for preclinical studies. Another example is EPPI-Reviewer ^12^, a tool widely used in clinical and social scientific evidence synthesis, which is the core evidence synthesis platform for the UK’s National Institute for Health and Care Excellence. The new online tool will be integrated within these two platforms and, since it is open source, it is available for integration within other systematic review platforms too.

The ultimate aim for the future would be “living” systematic reviews, which are updated constantly as new research evidence becomes available ^13^. Given the scarcity and expense of human input, the use of new technologies—including automation—is being evaluated for these types of reviews ^14^. Moreover, the human/machine axis may not be considered as binary opposites, as citizen science platforms, such as Cochrane Crowd, have shown that workflows can be developed that maximise the efficacy of human and machine contribution.

Unfortunately, the complete automation of outcome data extraction from graphs currently seems unlikely due to the varied nature of graphs and, as in most reviews, not every graph requires extraction, so human intelligence is required to decide which graph is the most relevant. However, for us to move towards goals of minimal human time to get maximum output, specifically for outcome measure extraction, we propose that further software development work be undertaken to support the automatic:

- identification of graph axes and their values, and optical character recognition to digitise text (e.g. axes labels), so a reviewer does not need to enter these manually ^15^
- recognition of figures that are potentially relevant for a research question
- recognition of figures that are definitely not relevant for a research question
- flagging of discrepancies between reviewers and identification of patterns within these, so that time is saved when resolving discrepancies.

### Limitations

There are several potential limitations to this evaluation. First, we are unlikely to have identified all graph types that are present in the clinical and preclinical literature. However, we believe that the graph types identified include most commonly used formats; other formats such as flow cytometry outputs are rarely extracted in the context of meta-analysis and so their omission is unlikely to have a major impact on our findings. This is supported by the observation that 89% of trial participants either agreed or strongly agreed that the online tool covered the most important graph types.

Second, this is a small study. We did not set out to show the superiority of the new, customised tool, and no conclusions of superiority should be drawn. However, we believe that it is reasonable to characterise the effectiveness of the tool as being promising.

Third, the extent to which the trial accurately reflected ‘real-life’ data extraction might be questioned, because in real-life, the reviewer would also be reading the rest of the paper, and maybe only extracting one time point from each graph and extracting other information such as group numbers or details of the paper. However, this trial aimed to separate the data extraction from this, so it could be analysed as a separate entity without other confounding aspects.

Finally, although not explicitly measured here, we observed that most data points that were extracted with sufficient accuracy using the graphical data extraction application had 100% of responses within the tolerable bounds; whereas in the current methods, even those that achieved sufficient accuracy often had responses outside of the tolerable bounds. Had we explored accuracy at the individual participant level, we would have likely seen even greater gains in accuracy in the graphical data extraction application condition.

## Conclusions

We have detailed here the motivation for, and development of, the customisation of two existing JavaScript libraries to create a new web browser-based tool to facilitate the extraction of quantitative data from graphs embedded in pdf files. We evaluated its utility in terms of its efficiency and accuracy, finding that it demonstrated non-inferiority compared to current practice in both dimensions. Our study suggests that the incorporation of this type of tool in online systematic review software would be beneficial in facilitating the production of accurate and timely evidence synthesis to improve decision-making.

## Data availability

Raw data associated with this study, including results of the survey, are available on Zenodo, DOI: https://doi.org/10.5281/zenodo.1482487 ^9^.

## Software availability

Source code available from: https://github.com/EPPI-Centre/Graph2Data.

Archived code at time of publication: https://doi.org/10.5281/zenodo.1484506 ^9^.

License: GNU Affero General Public License

## Supplementary material

**Supplementary File 1. Survey questions.**

**Supplementary File 2. Information sheet given to each participant during recruitment.**

**Supplementary File 3. Consent form for subjects taking part in the study.**

**Supplementary File 4. The tolerable bounds for determining accuracy of each data point, by graph.**

**Supplementary File 5. The graphs used for evaluation of the methods of data extraction.**

**Supplementary File 6. List of 34 pre-clinical studies.**

**Supplementary File 7. List of 12 public health studies.**
