# Supplementary material for: The development and evaluation of an online application to assist in the extraction of data from graphs for use in systematic reviews
Source: Wellcome Open Res. 2019 Mar 7;3:157. Originally published 2018 Dec 10. [Version 3] doi: 10.12688/wellcomeopenres.14738.3 (PMC6372928; doi:10.12688/wellcomeopenres.14738.3)
Supplement: Supplementary file 1 [file wellcomeopenres-3-16552-s0000.tgz › 1805b854-050a-4117-8ce6-ac96387fb057_Supp_file_1._Survey_questions.docx]

# Supplementary File 1. Survey questions

1. Name
2. Are you:
   1. Employed: Higher Education Institute
   2. Employed: Industry
   3. Employed: Governmental Body
   4. Student: Bachelors
   5. Student: Masters
   6. Student: Doctoral
   7. Other (free text box to specify)
3. What is your main discipline of work/study?
   1. Preclinical Science
   2. Clinical Science/ Medicine
   3. Statistics/ Mathematics
   4. Social Science
   5. Other (free text box to specify)
4. Please tick all the stages of a systematic review that you have performed, either in part or whole.
   1. Writing a Protocol
   2. Designing Search Terms
   3. Searching databases
   4. Screening for inclusion/exclusion
   5. Extracting meta-data (risk of bias items, intervention/ model details)
   6. Extracting outcome data
   7. Meta-analysis
   8. Write up
   9. None of the above
5. What tools to extract graphical data for systematic review and meta-analysis had you used before participating in this project? (Please tick all that apply)
   1. Universal Desktop Ruler
   2. In-built Adobe measuring tool
   3. Pencil, ruler and paper
   4. Web plot digitiser
   5. I have not extracted graphical data before
   6. Other (free text box to specify)
6. Which tool did you use for manual extraction in this trial? If you used more than one please select the one you most frequently used.
   1. Universal Desktop Ruler
   2. In-built Adobe measuring tool
   3. Pencil, ruler and paper
   4. Web plot digitiser
   5. Other (free text box to specify)
7. Please mark whether you agree or disagree with the following statements. (1=strongly disagree, 2= disagree, 3= neither agree not disagree, 4=agree, 5=strongly agree)
   1. The YouTube instruction video was useful
   2. The YouTube instruction video was comprehensive
   3. The online tool was easy to use
   4. The online tool got easier to use as I extracted from more graphs
   5. I think I got progressively faster using the tool
   6. The online tool had all the features I think it should have
   7. The online tool had all the features I think it should have
   8. Any further comments? (free text box to specify)
8. If you had to extract a third set of similar graphs using just one of the methods, current techniques or the new tool, which would you choose?
   1. Current techniques
   2. New online tool
9. Why would you choose the method selected above? (free text box)
