# Supplementary material for: The development and evaluation of an online application to assist in the extraction of data from graphs for use in systematic reviews
Source: Wellcome Open Res. 2019 Mar 7;3:157. Originally published 2018 Dec 10. [Version 3] doi: 10.12688/wellcomeopenres.14738.3 (PMC6372928; doi:10.12688/wellcomeopenres.14738.3)
Supplement: Supplementary file 2 [file wellcomeopenres-3-16552-s0001.tgz › 613db8a1-2543-419f-b81b-0e3f72c575f2_Supp_file_2._Information_sheet_given_to_each_participant_during_recruitment.docx]

# Supplementary file 2. Information sheet given to each participant during recruitment

**Who is conducting the research?**

My name is Fala Cramond from Imperial College London and I am inviting you to take part in our research project, ‘SLIM: Evaluation of computer-assistance in data extraction’. I am a research assistant specialising in preclinical systematic reviews, currently working under a joint Medical Research Council and Wellcome Trust grant named ‘SLIM’ (systematic living information machine). The purpose of the broader project is to develop and test the utility of machine-learning and text-mining tools in the systematic review process in preclinical (*in vivo*) domains, although we anticipate benefits for systematic reviewers across many disciplines. The principal investigators for this component of the project are Prof James Thomas (UCL) and Prof Andrew Rice (Imperial).

One of the stages of systematic review is the extraction of outcome measure data and this is one of the lengthiest components of a review. We have developed a tool to assist outcome extraction from plots and we need to evaluate this tool to see if it would be justified, feasible and beneficial to implement it on a wider scale.

I very much hope that you would like to take part. This information sheet will try to answer any questions you might have about the project, but please do not hesitate to contact me if there is anything else you would like to know.

**Why are we doing this research?**

We are doing this research to improve the quality and efficiency of preclinical systematic reviews, with anticipated benefits for systematic reviews across disciplines. Systematic reviews are a vital research tool, as evidence by their importance in clinical research domains, but the time they take to complete weakens their usefulness as they are often out of date shortly after being published. This is exacerbated by the exponentially increasing number of publications on databases such as PubMed.

Systematic reviews can generally be split into 6 stages: writing a protocol, searching, screening for inclusion/exclusion, extracting meta-data, extracting outcome measures and if appropriate, meta-analysis. The SLIM project has component sub-projects tackling all areas, and this component is specifically interested in extracting outcome data. This traditionally entails recording the data from a publication, which could be presented in a table, in a graph, or in the text, into a database and then this data will undergo meta-analysis. This is a lengthy and laborious process. Therefore, the purpose of this research is to investigate if the outcome extraction process can be facilitated.

This particular evaluation relates to a tool that we have developed to help with the extraction of outcome data from plots (e.g., bar charts, line graphs, etc.). As this process can be time-consuming and—potentially—error-prone, we want to assess whether using a computer-based tool can make the extraction from plots faster and more accurate.

**Why am I being invited to take part?**

You are being invited to take part because you are known to the investigators as having experience in or knowledge of systematic review methods. Therefore, we hope this research is of interest to you because it is applicable to your current role, studies or existing knowledge of systematic review.

**What will happen if I choose to take part?**

The trial will compare the new tools performance to current methods of outcome data extraction. Therefore, you will be asked to extract (fictitious) outcome data from two sets of graphs, one set using the new tool and one set using your current preferred methods.

If you choose to take part the following will happen:

1. You will be asked to register for two online platforms. You will be given a log in that will be required when you use either platform
2. You will then be sent two PDF files. Each of these files is a set of 24 fictitious graphs of different types (bar chart, line graph, etc)
3. You will be instructed to extract the data from one of these files these graphs using one of the methods
4. After completing the first set of graphs, you will extract the data from the remaining set of graphs using the other method
5. After you have completed the second PDF, you will be sent a link to an online questionnaire which you will be asked to fill in. This anonymous questionnaire will ask questions about your views and experiences of the two methods of extraction

Overall, we expect the whole task will take less than two hours. You will be able to stop the task and return to it at a later time/date without this affecting your results, i.e. you do not need to complete it in one sitting.

All of this can be done remotely and at a time that suits you. Data collection will be done automatically as the two online platforms will have this built in. We hope that you will complete all activities, however, you are able to withdraw at any point during the study with no negative repercussions and any data you have submitted up to that point will be deleted.

**Will anyone know I have been involved?**

No, no one outside the key investigators will know you have taken part. All contributions will be anonymised before dissemination and will be untraceable back to you.

**Could there be problems for me if I take part?**

We do not expect there to be any major problems for you if you take part. We will not be asking for any personal or sensitive information. However, as participation will require some of your time, this could lead to minor stress meeting other deadlines. To counteract this there is a generous time frame in which you should complete the task and you are entitled to stop at any point.

**What will happen to the results of the research?**

Whilst we do not anticipate the results of this research to be published in an academic journal, the results may be posted on the website(s) of the collaborators in the project. Results will be circulated amongst collaborators and funders of the current project and may be used as support for future funding.

Participants will be sent a copy of the results.

Data will be stored on a secure server system, password protected and only accessible to the key investigators in this project. Data will be retained until future activities that may require the data have been discussed and confirmed.

**Do I have to take part?**

It is entirely up to you whether or not you choose to take part. We hope that if you do choose to be involved then you will find it a valuable experience.

Choosing not to participate, or changing your mind and withdrawing during the study, will have no negative repercussions, neither academically nor professionally.

**Thank you very much for taking the time to read this information sheet.**

**If you would like to be involved, please complete the following consent form and return to**

Fala Cramond ([f.cramond@imperial.ac.uk](mailto:f.cramond@imperial.ac.uk), 4^th^ Floor Pain Research Group, Imperial College London, Chelsea and Westminster Hospital, 369 Fulham Road, London, SW10 9NH)

**If you have any further questions before you decide whether to take part, you can email me at** [**f.cramond@imperial.ac.uk,**](mailto:f.cramond@imperial.ac.uk) **or call me on 07530765965 or 02087468424. Alternatively, you can contact the principal investigator overseeing this evaluation, Professor James Thomas, at** [**james.thomas@ucl.ac.uk.**](mailto:james.thomas@ucl.ac.uk)

**This project has been reviewed and approved by the UCL IOE Research Ethics Committee**
