# Supplementary material for: The development and evaluation of an online application to assist in the extraction of data from graphs for use in systematic reviews
Source: Wellcome Open Res. 2019 Mar 7;3:157. Originally published 2018 Dec 10. [Version 3] doi: 10.12688/wellcomeopenres.14738.3 (PMC6372928; doi:10.12688/wellcomeopenres.14738.3)
Supplement: Supplementary file 3 [file wellcomeopenres-3-16552-s0002.tgz › fa39e517-6b5b-4369-9190-c7044528f206_Supp_file_3._Consent_form.docx]

# Supplementary file 3. Consent Form

If you are happy to participate, please complete this consent form and return to Fala Cramond ([f.cramond@imperial.ac.uk](mailto:f.cramond@imperial.ac.uk), 4th Floor Pain Research Group, Imperial College London, Chelsea and Westminster Hospital, 369 Fulham Road, London, SW10 9NH)

- I have read and understood the information leaflet about the research
- I agree to undertake the evaluation and fill in the subsequent questionnaire
- I understand that if any of my words are used in reports or presentations they will not be attributed or traceable back to me
- I understand that participation is voluntary
- I understand that I can withdraw from the project at any time, and that if I choose to do this, any data I have contributed will not be used
- I understand that I can contact Fala Cramond at any time
- I understand that the results will be shared with our collaborators and funding bodies
