# Supplementary material for: The development and evaluation of an online application to assist in the extraction of data from graphs for use in systematic reviews
Source: Wellcome Open Res. 2019 Mar 7;3:157. Originally published 2018 Dec 10. [Version 3] doi: 10.12688/wellcomeopenres.14738.3 (PMC6372928; doi:10.12688/wellcomeopenres.14738.3)
Supplement: Supplementary file 4 [file wellcomeopenres-3-16552-s0003.tgz › 31d3f392-6a89-413e-a14f-fed08c2ded43_Supp_file_4._Tolerable_bounds_for_determining_accuracy_of_each_data_point,_by_graph.docx]

# Supplementary file 4. Tolerable bounds for determining accuracy of each data point, by graph

| **Graph number** | **Bound value** |
| --- | --- |
| 1 | 1 |
| 2 | 0.1 |
| 3 | 0.5 |
| 4 | 1 |
| 5 | 1 |
| 6 | 0.1 |
| 7 | 1 |
| 8 | 0.025 |
| 9 | 0.1 |
| 10 | 0.5 |
| 11 | 0.1 |
| 12 | 1 |
| 13 | 0.25 |
| 14 | 2.5 |
| 15 | 0.25 |
| 16 | 1 |
| 17 | 0.1 |
| 18 | 0.05 |
| 19 | 100 |
| 20 | 2.5 |
| 21 | 1 |
| 22 | 0.25 |
| 23 | 0.1 |
