# Supplementary figures and images for: The development and evaluation of an online application to assist in the extraction of data from graphs for use in systematic reviews
Source: Wellcome Open Res. 2019 Mar 7;3:157. Originally published 2018 Dec 10. [Version 3] doi: 10.12688/wellcomeopenres.14738.3 (PMC6372928; doi:10.12688/wellcomeopenres.14738.3)

# Supplementary file 5. Graphs included in the evaluation


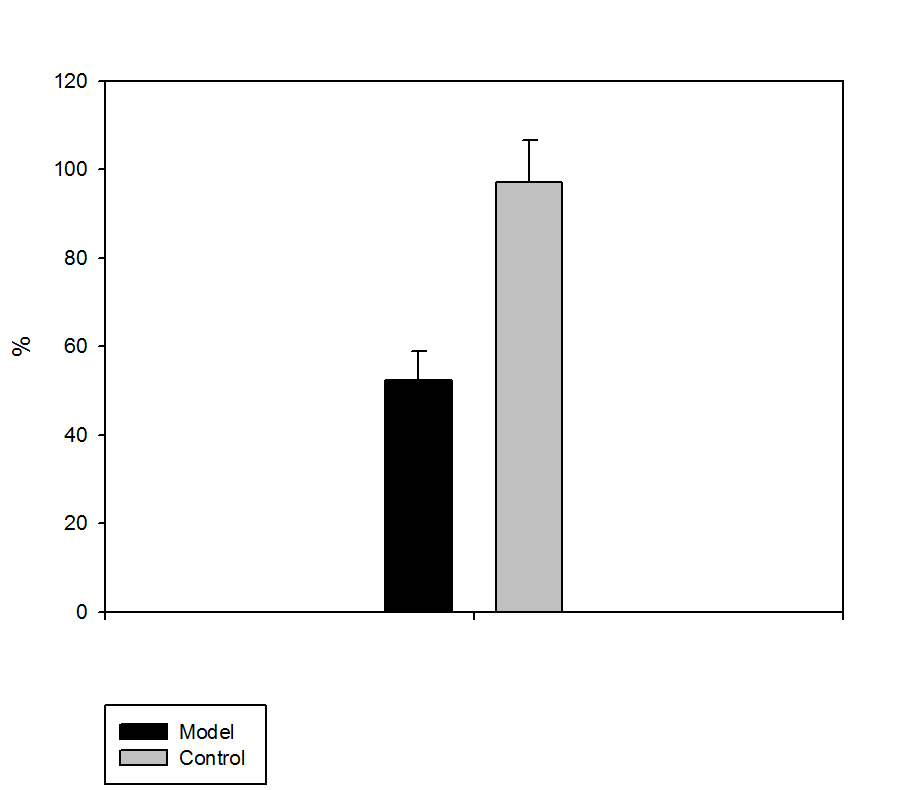

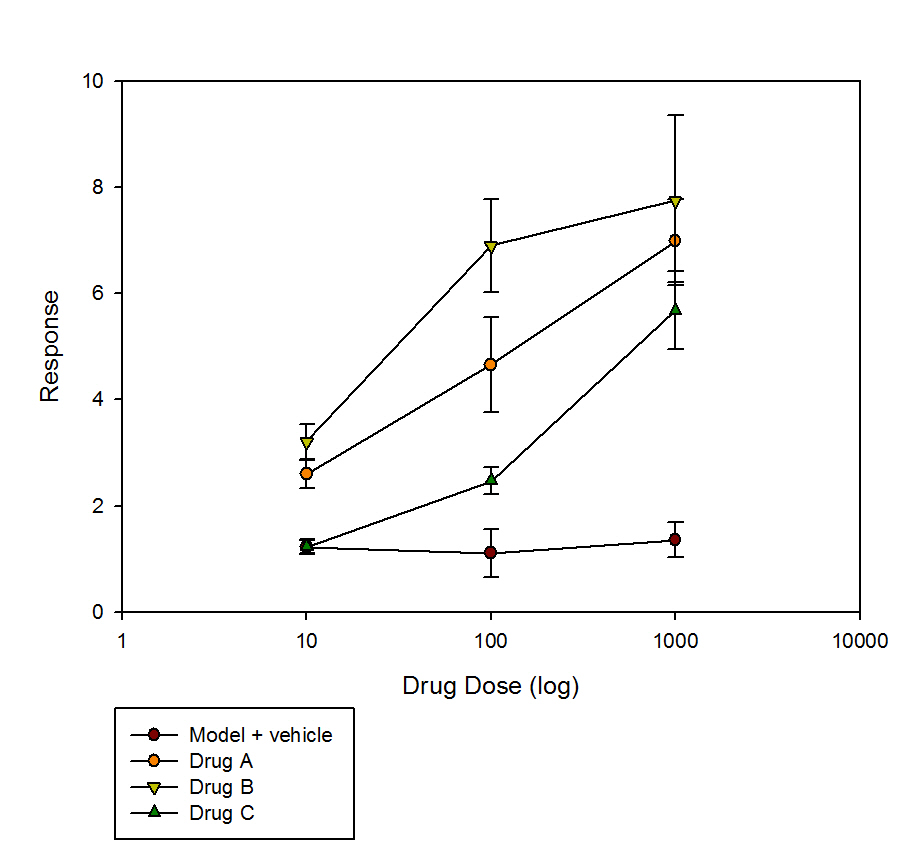

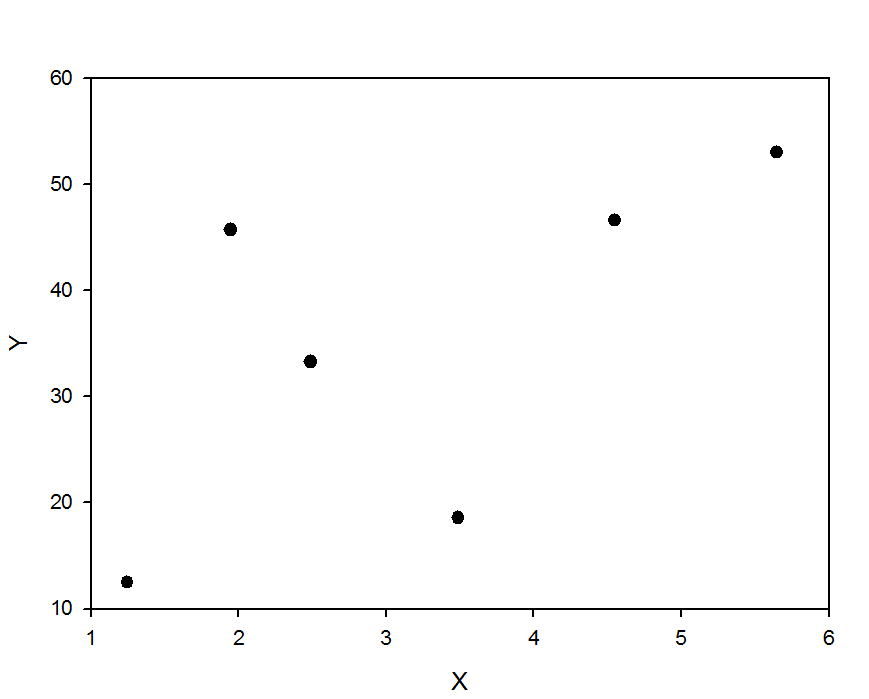

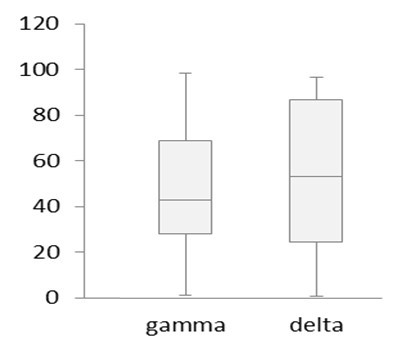

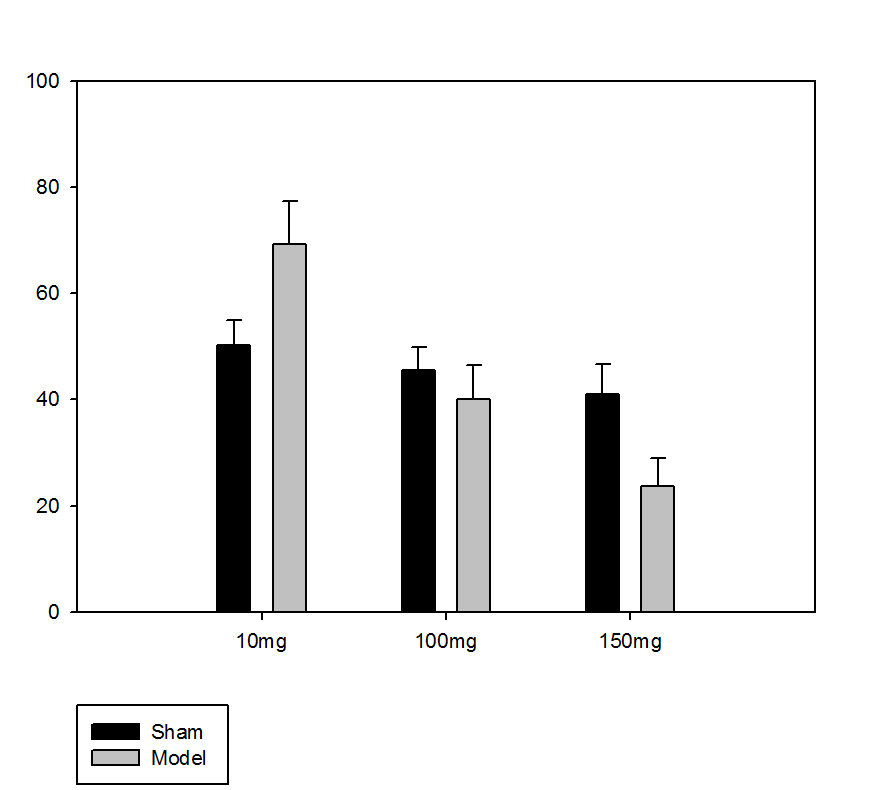

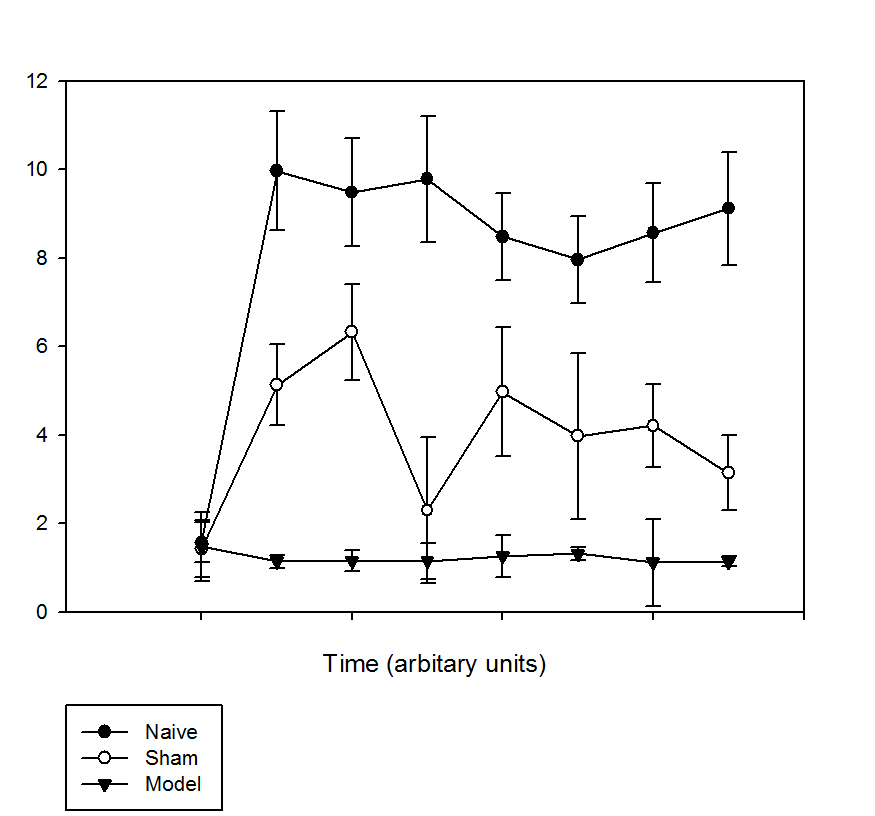

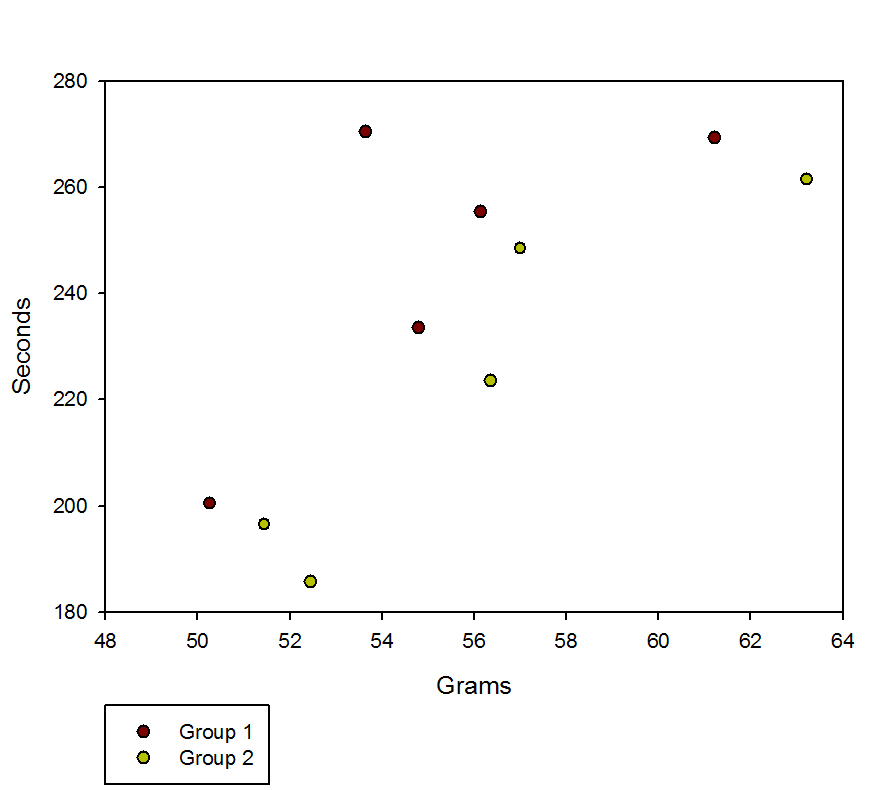

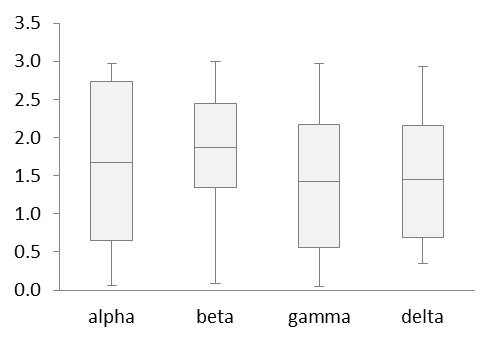

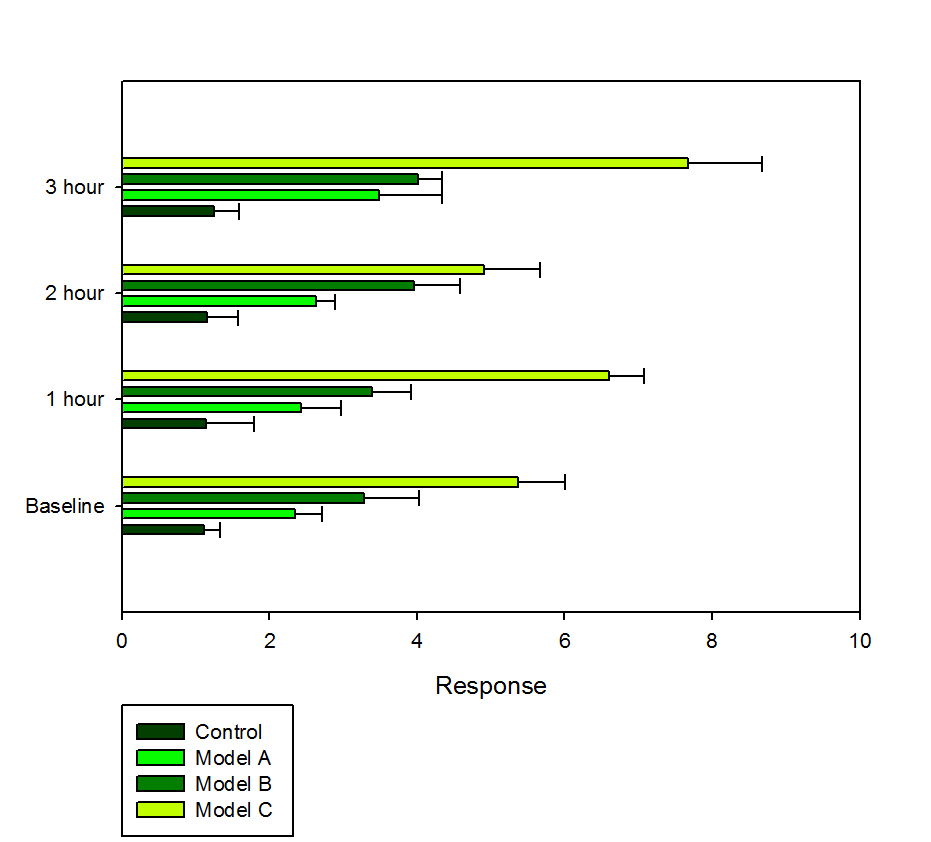

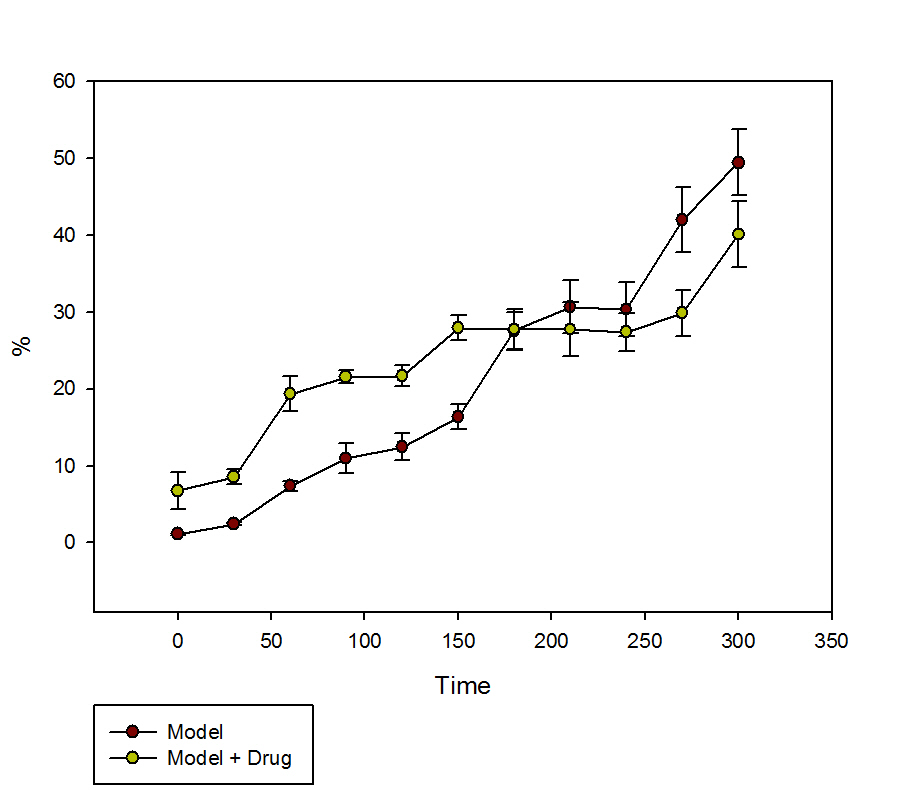

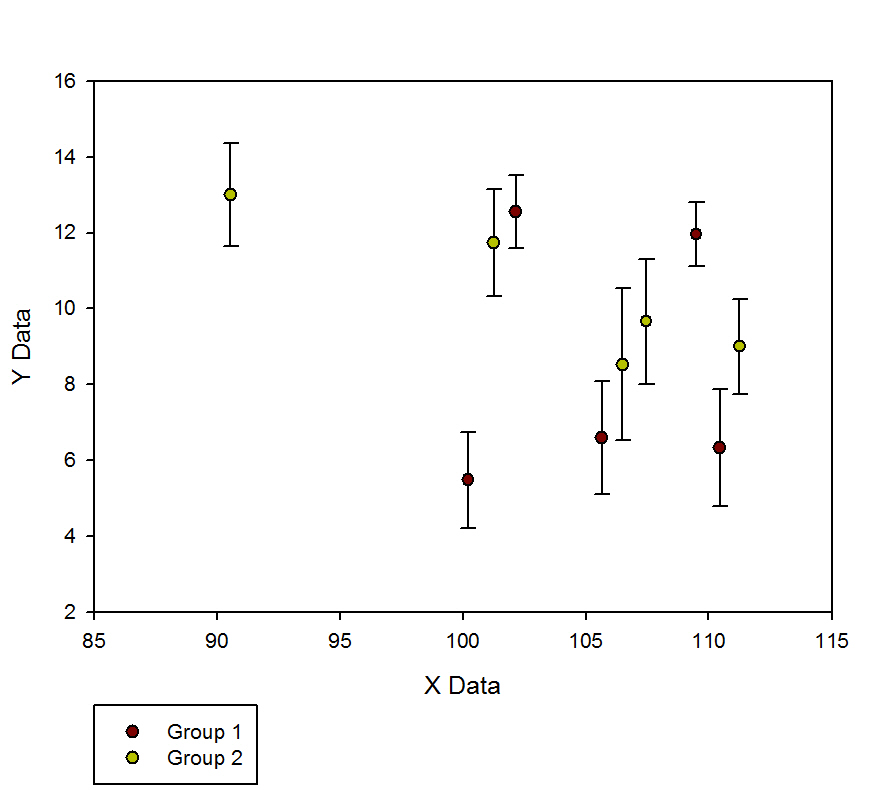

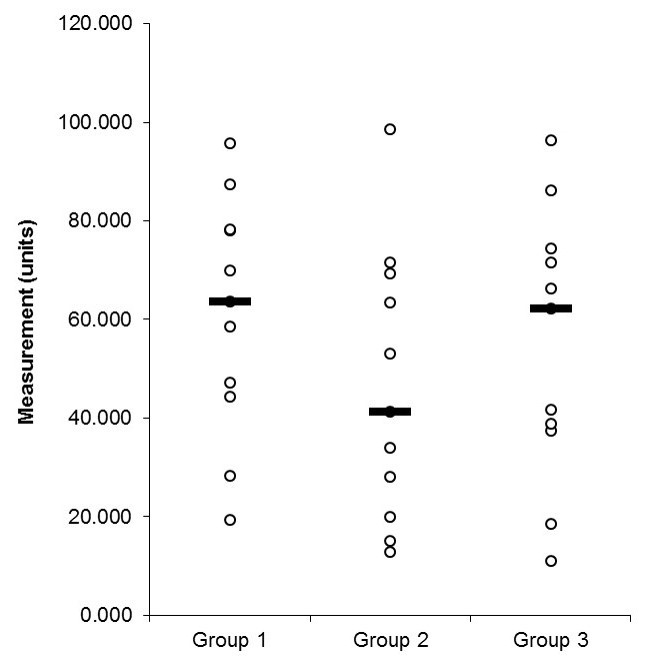

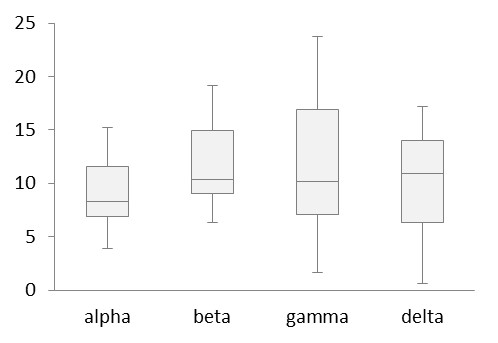

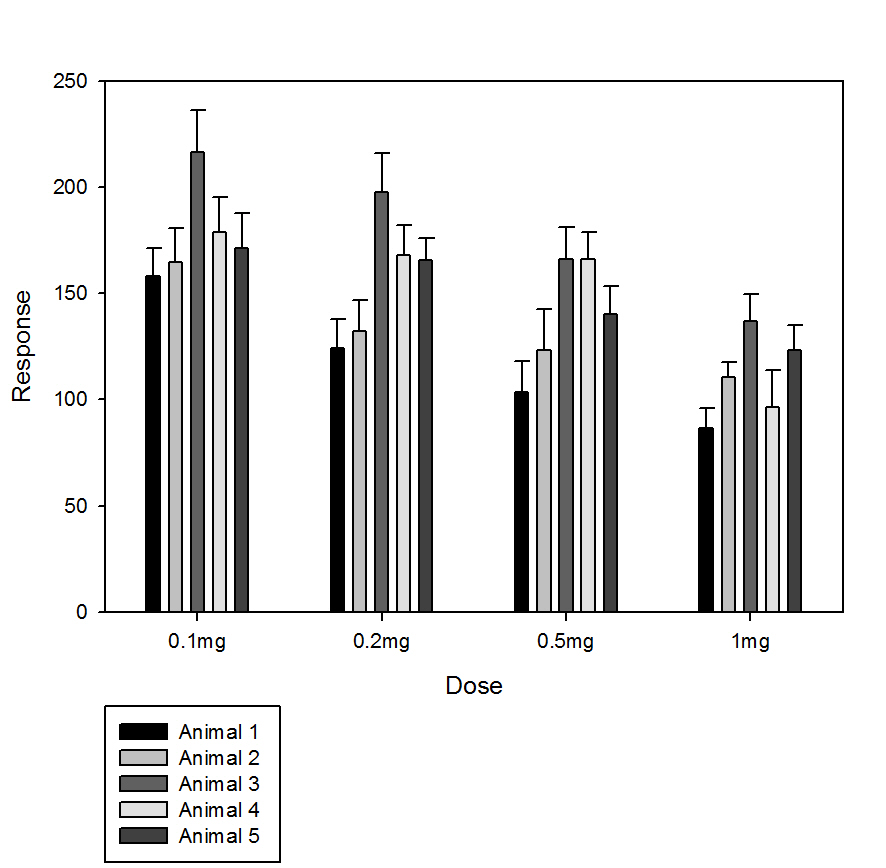

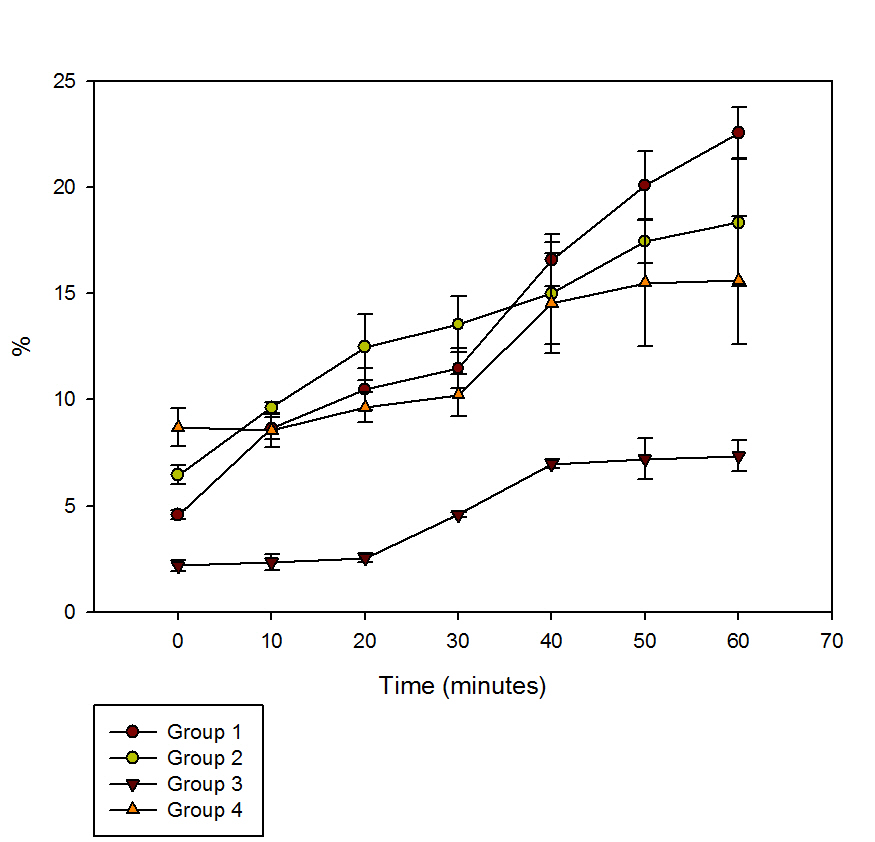

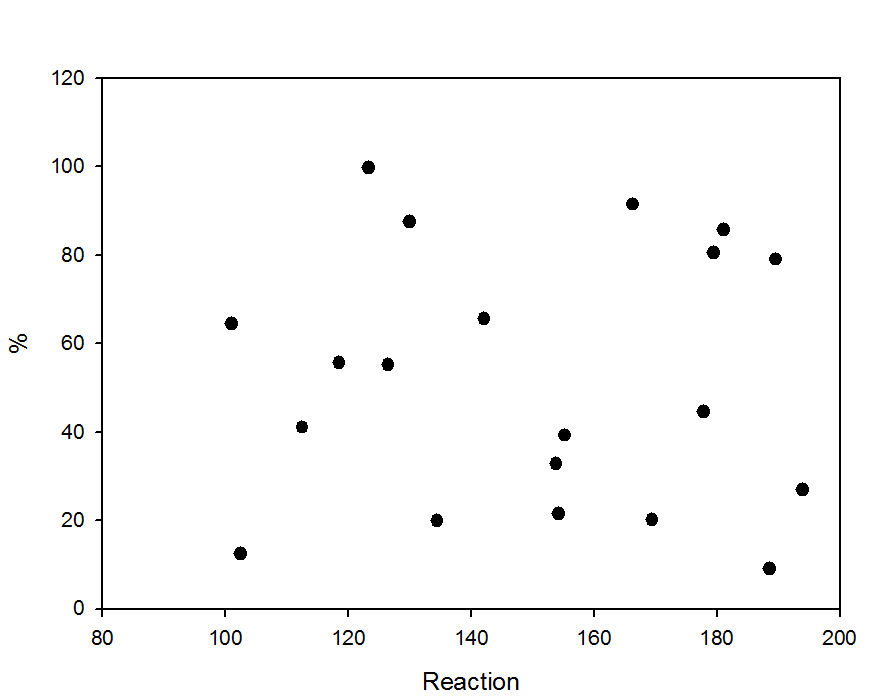

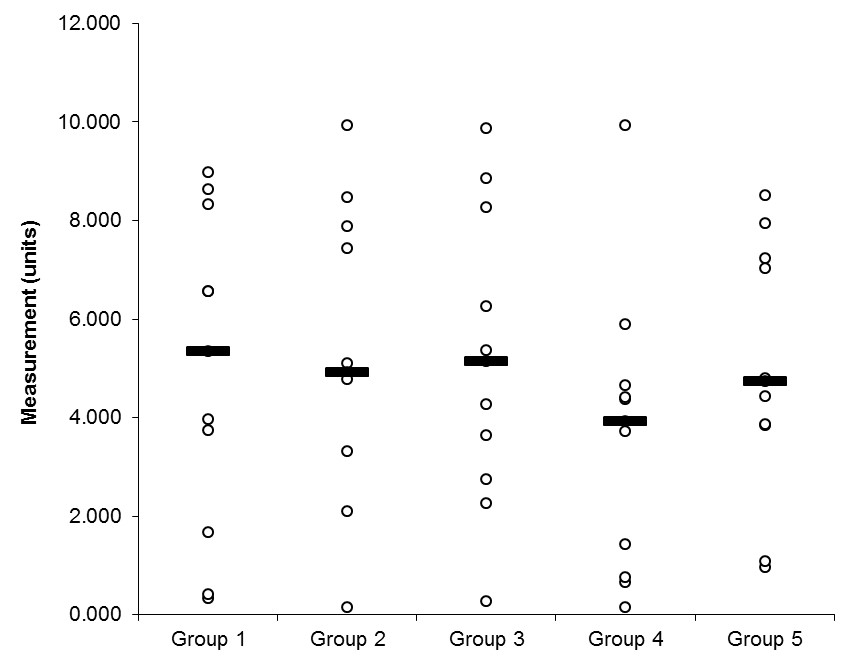

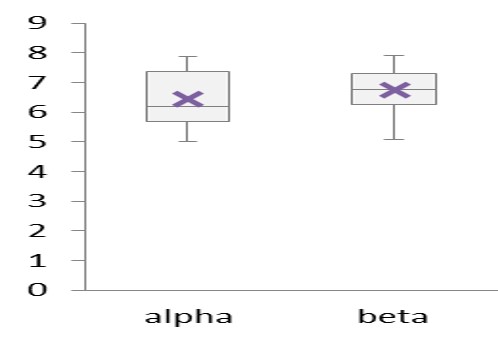

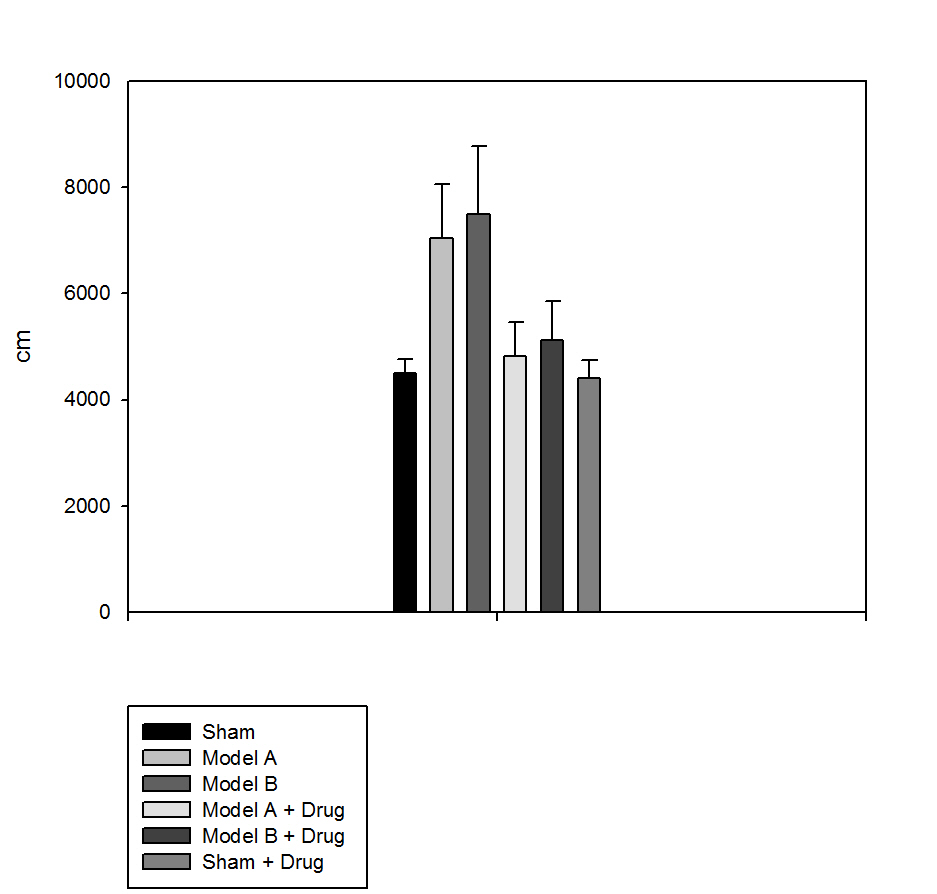


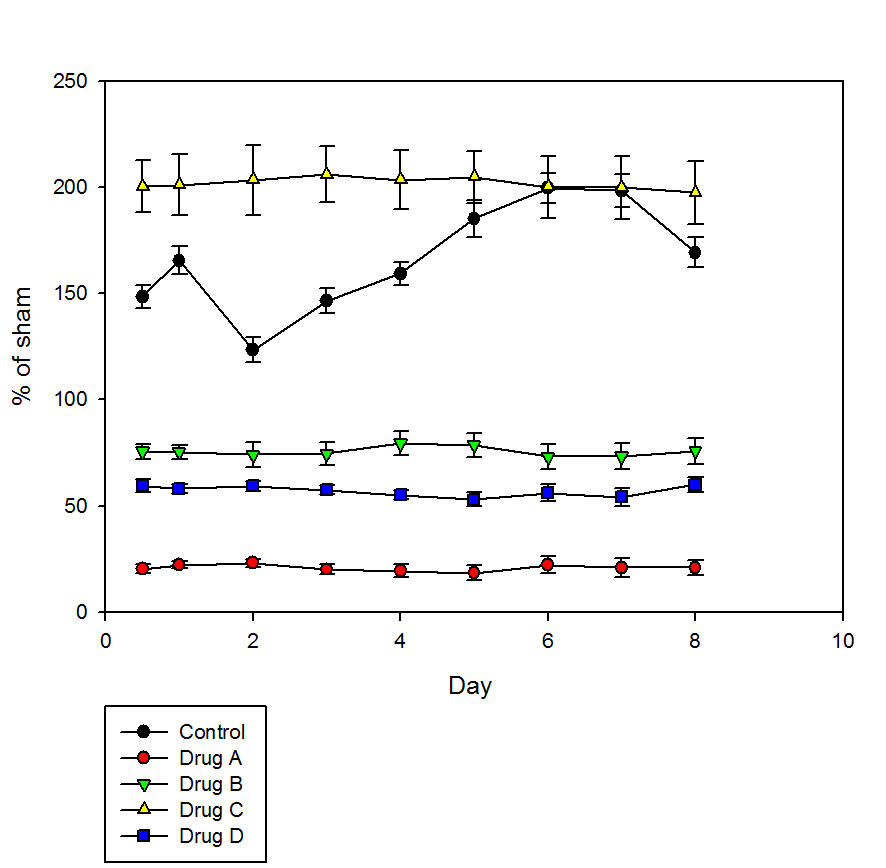


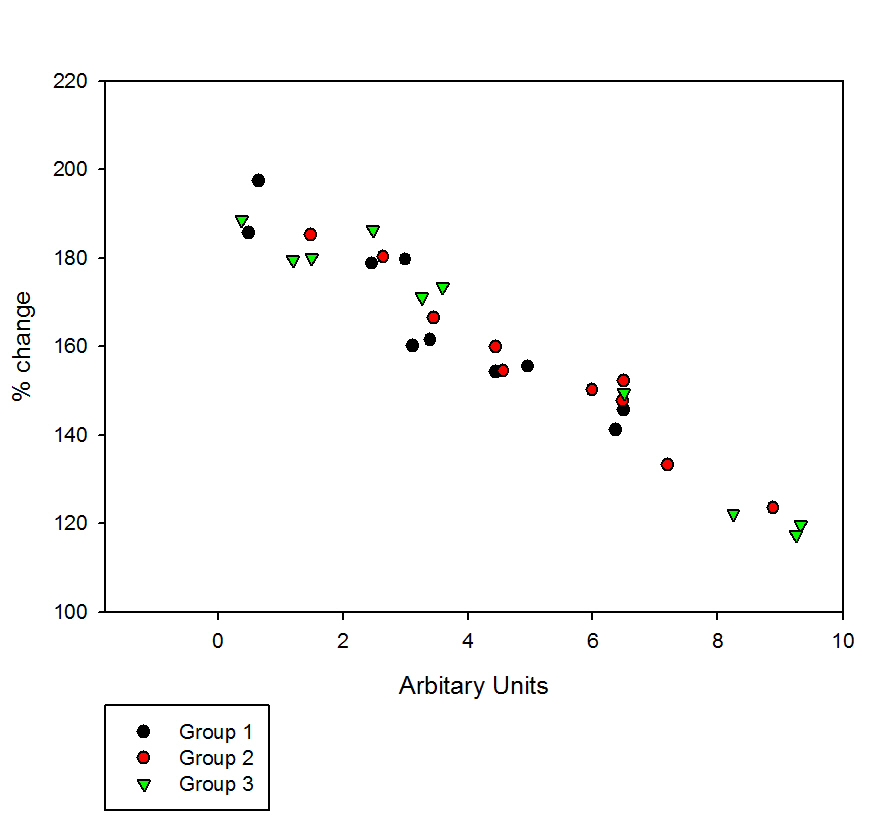

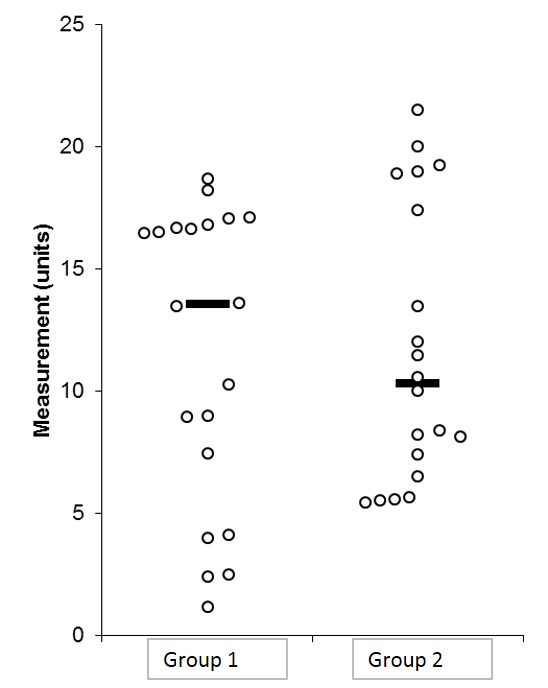

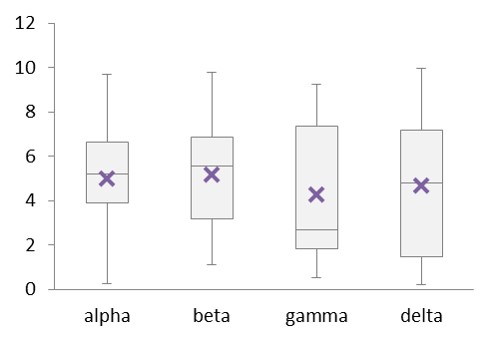

Supplement: Supplementary file 5 [file wellcomeopenres-3-16552-s0004.tgz › 8c0a676b-a8aa-49cf-8fd5-e5d201f377a2_Supp_file_5._Graphs_included_in_the_evaluation.docx]
