# Supplementary material for: The development and evaluation of an online application to assist in the extraction of data from graphs for use in systematic reviews
Source: Wellcome Open Res. 2019 Mar 7;3:157. Originally published 2018 Dec 10. [Version 3] doi: 10.12688/wellcomeopenres.14738.3 (PMC6372928; doi:10.12688/wellcomeopenres.14738.3)
Supplement: Supplementary file 6 [file wellcomeopenres-3-16552-s0005.tgz › 7acd0b73-905b-4355-a378-1fe4b702099e_Supplementary_File_6._List_of_34_pre-clinical_studies.docx]

# List of 34 pre-clinical studies

Bishay P, Schmidt H, Marian C, Haussler C, Wijnvoord N, Ziebell S, Metzner J, Koch M, Myrczek T, Bechmann I, Kuner R, Costigan M, Dehghani F, Geisslinger G, and Tegeder I. (2010). R-Flurbiprofen Reduces Neuropathic Pain in Rodents by Restoring Endogenous Cannabinoids. *PLOS one*, , pp.e10628.

Casals-Díaz L, Vivó M, and Navarro X. (2009). Nociceptive responses and spinal plastic changes of afferent C-fibers in three neuropathic pain models induced by sciatic nerve injury in the rat. *Experimental Neurology*, 217, pp.84-95.

Christopher M Peters, Juan Miguel Jimenez-Andrade, Beth M Jonas, Molly A Sevcik, Nathan J Koewler, Joseph R Ghilardi, Gilbert Y Wong, and Patrick W Mantyh. (2007). Intravenous paclitaxel administration in the rat induces a peripheral sensory neuropathy characterized by macrophage infiltration and injury to sensory neurons and their supporting cells. *Experimental Neurology*, , pp.42–54.

Erschbamer M, Pernold K, and Olson L. (2007). Inhibiting Epidermal Growth Factor Receptor Improves Structural, Locomotor, Sensory, and Bladder Recovery from Experimental Spinal Cord Injury. *The Journal of Neuroscience*, , pp.6428–6435.

F S Hasnie, J Breuer, S Parker, V Wallace, J Blackbeard, I Lever, P R Kinchington, A H Dickensond, T Pheby, and A S C. Rice. (2007). Further characterisation of a rat model of varicella zoster virus (VZV)-associated pain: relationship between mechanical hypersensitivity and anxiety-related behaviour; and the influence of analgesic drugs. *Neuroscience.*, , pp.1495–1508..

Fabien Marchand, Christoforos Tsantoulas, Dalbinder Singh, John Grist, Anna K Clark, Elizabeth J Bradbury, and Stephen B McMahon. (2009). Effects of Etanercept and Minocycline in a rat model of spinal cord injury. *European Journal of Pain*, , pp.673–681.

Garry EM, Delaney A, Anderson HA, Sirinathsinghji EC, Clapp RH, Martin WJ, Kinchington PR, Krah DL, Abbadie C, and Fleetwood-Walker SM. (2005). Varicella zoster virus induces neuropathic changes in rat dorsal root ganglia and behavioral reflex sensitisation that is attenuated by gabapentin or sodium channel blocking drugs. *Pain*, , pp. 97–111.

Grelik C, Bennett GJ, and Ribeiro-da-Silva A. (2005). Autonomic fibre sprouting and changes in nociceptive sensory innervation in the rat lower lip skin following chronic constriction injury. *European Journal of Neuroscience*, , pp.2475–2487.

Hama A, and Sagen J. (2007). Antinociceptive effect of cannabinoid agonist WIN 55,212–2 in rats with a spinal cord injury. *Exp Neurol*, , pp.454–457.

Haraguchi K, Kawamoto A, Isami K, Maeda S, Kusano A, Asakura K, Shirakawa H, Mori Y, Nakagawa T, and Kaneko S. (2012). TRPM2 Contributes to Inflammatory and Neuropathic Pain through the Aggravation of Pronociceptive Inflammatory Responses in Mice. *The Journal of Neuroscience*, , pp.3931–3941.

Hofstetter CP, Schweinhardt P, Klason T, Olson L, and Spenger C. (2003). Numb rats walk – a behavioural and fMRI comparison of mild and moderate spinal cord injury . *European Journal of Neuroscience*, , pp.3061-3068.

Horiuchi H, Ogata T, Morino T, Takeba J, and Yamamoto H. (2003). S erotonergic signaling inhibits hyperalgesia induced by spinal cord damage. *Brain Research*, , pp.312–320.

Huang W, Calvo M, Karu K, Olausen HR, Bathgate G, Okuse K, Bennett DL, and Rice ASC. (2013). A clinically relevant rodent model of the HIV antiretroviral drug stavudine induced painful peripheral neuropathy. *Pain*, , pp.560-575.

Imbe H, Abe T, Okamoto K, Sato M, Ito H, Kumabe S, and Senba E. (2004). Increase of galanin-like immunoreactivity in rat hypothalamic arcuate neurons after peripheral nerve injury. *Neuroscience Letters*, , pp.102–106.

Kelli A Sullivan, John M Hayes, Timothy D Wiggin, Carey Backus, Sang Su Oh, Stephen I Lentz, Frank Brosius III, and Eva L Feldman. (2007). Mouse Models of Diabetic Neuropathy. *Neurobiol Dis.*, , pp.276–285.

Keswani SC, 1 Jack C, 1 Zhou C, 1 , Ho A¨ke1, and 2 . (2006). Establishment of a Rodent Model of HIV-Associated Sensory Neuropathy. *The Journal of Neuroscience*, , pp.10299 –10304.

Lee MJ, Shin TJ, Lee JE, Choo H, Koh HY, Chung HJ, Pae AN, Lee SC, and Kim HJ. (2010). KST5468, a new T-type calcium channel antagonist, has an antinociceptive effect on inflammatory and neuropathic pain models. *Pharmacology, and Biochemistry and Behavior*, , pp.198–204.

Megan S Johnson, Janelle M Ryals, and Douglas E Wright. (2008). Early loss of peptidergic intraepidermal nerve fibers in an STZ induced mouse model of insensate diabetic neuropathy. *Pain.*, , pp.35–47..

Momin A, Cadiou H, Mason A, and McNaughton PA. (2008). Role of the hyperpolarization-activated current Ih in somatosensory neurons. *J Physiol*, , pp.5911–5929.

Nakajima K, Obata H, Iriuchijima N, and Saito S. (2012). An increase in spinal cord noradrenaline is a major contributor to the antihyperalgesic effect of antidepressants after peripheral nerve injury in the rat. *PAIN*, , pp.990-997.

Polomano RC, Mannes AJ, Clark US, and Bennett GJ. (2001). A painful peripheral neuropathy in the rat produced by the chemotherapeutic drug, paclitaxel. *Pain*, , pp.293–304.

R R SMITH, S MARTIN-SCHILD, A J KASTIN, and J E ZADINA. (2001). DECREASES IN ENDOMORPHIN-2-LIKE IMMUNOREACTIVITY CONCOMITANT WITH CHRONIC PAIN AFTER NERVE INJURY. *Neuroscience*, , pp.773-778.

Raghavendra V, Rutkowski MD, and DeLeo JA. (2002). The Role of Spinal Neuroimmune Activation in Morphine Tolerance/ Hyperalgesia in Neuropathic and Sham-Operated Rats. *The Journal of Neuroscience*, , pp.9980–9989.

Raygude KS, Kandhare AD, Ghosh P, Ghule AE, and Bodhankar SL. (2012). Evaluation of ameliorative effect of quercetin in experimental model of alcoholic neuropathy in rats. *Inflammopharmacol*, , pp.331–341.

Romero-Sandoval A, Nutile-McMenemy N, and DeLeo JA. (2008). Spinal Microglial and Perivascular Cell Cannabinoid Receptor Type 2 Activation Reduces Behavioral Hypersensitivity without Tolerance after Peripheral Nerve Injury. *Anesthesiology*, , pp.722–734.

Seinoa H, Seoa K, Maedab T, and Someyaa T. (2009). Behavioural and histological observations of sensory impairment caused by tight ligation of the trigeminal nerve in mice. *Journal of Neuroscience Methods*, , pp. 67–72.

Shen Y, Zhang Z-J, Zhu M-D, Jiang B-C, Yanga T, and Gao Y-J. (2015). Exogenous induction of HO-1 alleviates vincristine-induced neuropathic pain by reducing spinal glial activation in mice. *Neurobiology of Disease*, , pp.100–110.

Shortland PJ, Baytug B, Krzyzanowska A, McMahon SB, Priestley JV, and Averill S. (2006). ATF3 expression in L4 dorsal root ganglion neurons after L5 spinal nerve transection. *European Journal of Neuroscience*, , pp.365–373 .

Shumilla JA, Liron T, Mochly-Rosen D, Kendig JJ, and Sweitzer SM. (2005). Ethanol Withdrawal–Associated Allodynia and Hyperalgesia: Age-Dependent Regulation by Protein Kinase C. *The Journal of Pain*, , pp.535-549.

Symons KT, Nguyen PM, Massari ME, Anzola JV, Staszewski LM, Wang L, Yazdani N, Dorow S, Muhammad J, Sablad M, Rozenkrants N, Bonefous C, Payne JE, Rix PJ, Shiau AK, Noble SA, Smith ND, Hassig CA, Zhang Y, and Rao TS. (2011). Pharmacological Characterization of KLYP961, a Dual Inhibitor of Inducible and Neuronal Nitric-Oxide Synthases. *THE JOURNAL OF PHARMACOLOGY AND EXPERIMENTAL THERAPEUTICS*, , pp.468-478.

Wallace VCJ, Blackbeard J, Segerdahl AD, Hasnie F, Pheby T, McMahon SB, and Rice ASC. (2007). Characterization of rodent models of HIV-gp120 and anti-retroviral-associated neuropathic pain. *Brain.*, , pp.2688–2702.

Weng HR, Cordella JV, and Dougherty PM. ( 2). Changes in sensory processing in the spinal dorsal horn accompany vincristine-induced hyperalgesia and allodynia. *Pain*, , pp.131–138.

Xiao WH, Zheng FY, Bennett GJ, Bordet T, and Pruss RM. (2009). Olesoxime (cholest-4-en-3-one, oxime): Analgesic and neuroprotective effects in a rat model of painful peripheral neuropathy produced by the chemotherapeutic agent, paclitaxel. *Pain*, , pp.202-209.

Yeo J-H, Yoon S-Y, Kim S-J, Oh S-B, Lee J-H, Beitz A-J, and Roh D-H. (2016). Clonidine, an alpha-2 adrenoceptor agonist relieves mechanical allodynia in oxaliplatin-induced neuropathic mice; potentiation by spinal p38 MAPK inhibition without motor dysfunction and hypotension. *Int. J. Cancer*, , pp.2466–2476.
