# Supplementary material for: The development and evaluation of an online application to assist in the extraction of data from graphs for use in systematic reviews
Source: Wellcome Open Res. 2019 Mar 7;3:157. Originally published 2018 Dec 10. [Version 3] doi: 10.12688/wellcomeopenres.14738.3 (PMC6372928; doi:10.12688/wellcomeopenres.14738.3)
Supplement: Supplementary file 7 [file wellcomeopenres-3-16552-s0006.tgz › 4394368e-e912-48b1-a363-920cbe58d2fb_Supplementary_File_7._List_of_12_public_health_studies.docx]

# List of 12 public health studies

Baruth Meghan, Wilcox Sara, Blair Steve, Hooker Steve, Hussey Jim, and Saunders Ruth. (2010). Psychosocial mediators of a faith-based physical activity intervention: implications and lessons learned from null findings. *Health Education Research*, 25(4), pp.645-655.

Brandon Thomas H. PhD, Simmons Vani Nath PhD, Meade Cathy D. R. N. PhD, Quinn Gwendolyn P. PhD, Khoury Elena N. Lopez PhD, Sutton Steven K. PhD, and Lee Ji-Hyun DrPH. (2012). Self-Help Booklets for Preventing Postpartum Smoking Relapse: A Randomized Trial. *American Journal of Public Health*, 102(11), pp.2109-2115.

Curnow M M, Pine C M, Burnside G, Nicholson J A, Chesters R K, and Huntington E. (2002). A randomised controlled trial of the efficacy of supervised toothbrushing in high-caries-risk children. *Caries Res*, 36(4), pp.294-300.

Johnson Z, Howell F, and Molloy B. (1993). Community mothers' programme: randomised controlled trial of non-professional intervention in parenting. *British Medical Journal*, 306(6890), pp.1449-1452.

Julnes George, Konefal Margaret, Pindur Wolfgang, and Kim Pan. (1994). Community-based perinatal care for disadvantaged adolescents: Evaluation of the resource mothers program. *Journal of Community Health*, 19(1), pp.41-53.

Mendoza Jason A, Levinger David D, and Johnston Brian D. (2009). Pilot evaluation of a walking school bus program in a low-income, urban community. *BMC Public Health*, 9(1), pp.122.

Nguyen Tung T, Le Gem, Nguyen Thoa, Le Khanh, Lai Ky, Gildengorin Ginny, Tsoh Janice, Bui-Tong Ngoc, and McPhee Stephen J. (2009). Breast Cancer Screening Among Vietnamese Americans: A Randomized Controlled Trial of Lay Health Worker Outreach. *American Journal of Preventive Medicine*, 37(4), pp.306-313.

Pollak Kathryn I, Oncken Cheryl A, Lipkus Isaac M, Lyna Pauline, Swamy Geeta K, Pletsch Pamela K, Peterson Bercedis L, Heine R Phillips, Namenek Brouwer, Rebecca J, Fish Laura, and Myers Evan R. (2007). Nicotine Replacement and Behavioral Therapy for Smoking Cessation in Pregnancy. *American journal of preventive medicine*, 33(4), pp.297-305.

Reijneveld S A, Westhoff M H, and Hopman-Rock M. (2003). Promotion of health and physical activity improves the mental health of elderly immigrants: results of a group randomised controlled trial among Turkish immigrants in the Netherlands aged 45 and over. *Journal of Epidemiology and Community Health*, 57(6), pp.405-411.

Rosser B R. Simon, Bockting Walter O, Rugg Deborah L, Robinson Beatrice Bean E, Ross Michael W, Bauer Greta R, and Coleman Eli. (2002). A Randomized Controlled Intervention Trial of a Sexual Health Approach to Long-Term HIV Risk Reduction for Men Who Have Sex with Men: Effects of the Intervention on Unsafe Sexual Behavior. *AIDS Education and Prevention*, 14(3 Supplement), pp.59-71.

Stanton Bonita, Harris Carole, Cottrell Lesley, Li Xiaoming, Gibson Catherine, Guo Jiantong, Pack Robert, Galbraith Jennifer, Pendleton Sara, Wu Ying, Burns James, Cole Matthew, and Marshall Sharon. (2006). Trial of an urban adolescent sexual risk-reduction intervention for rural youth: a promising but imperfect fit. *Journal of Adolescent Health*, 38(1), pp.55.e25-55.e36.

Winkleby M A, Feighery E, Dunn M, Kole S, Ahn D, and Killen J D. (2004). Effects of an advocacy intervention to reduce smoking among teenagers. *Archives of Pediatrics & Adolescent Medicine*, 158(3), pp.269-275.
