# Supplementary material for: The development and evaluation of an online application to assist in the extraction of data from graphs for use in systematic reviews
Source: Wellcome Open Res. 2019 Mar 7;3:157. Originally published 2018 Dec 10. [Version 3] doi: 10.12688/wellcomeopenres.14738.3 (PMC6372928; doi:10.12688/wellcomeopenres.14738.3)
Supplement: Peer review history

# Peer review: Reviewer response for version 2

**DOI:** 10.21956/wellcomeopenres.16420.r34676

**Published:** 2019-02-14

**Authors:** Chris Marshall

**Affiliations:** 1 Institute of Health and Society, Newcastle University, Newcastle, UK

Thank you to the authors for addressing my initial comments so comprehensively.  The extra detail and clarity concerning the aims, objectives and methods is excellent... I get it now!  My only other minor comment concerns a couple of lines in the final paragraph of Page 6:

  "a prototype workflow designed specifically around the needs of systematic reviewers"

 "The aim was not create a new tool that was ready for widespread deployment but to inform future development decisions, based on the evaluation, as to the utility of integrating such a tool in systematic review software"

 My comment is really just to say that I think these bits are great and cut right to the heart of the paper and what the work aimed to achieve. Perhaps these lines could be brought forward or integrated into the 'Research aim' section at the beginning of the methods?

 Valuable paper in this space I think, as we need more evaluations of systematic review tools!

I have read this submission. I believe that I have an appropriate level of expertise to confirm that it is of an acceptable scientific standard.

# Response

**Published:** 2019-03-02

**Authors:** James Thomas

**Affiliations:** University College London, UK

We would like to thank Chris Marshall for his second review, and are pleased that our revisions have addressed his concerns. We have inserted "The aim was not create a new tool that was ready for widespread deployment but to inform future development decisions, based on the evaluation, as to the utility of integrating such a tool in systematic review software” into the ‘aims’ section as suggested, to ensure our evaluation focus is clear at the start. We have also made a number of other amendments to improve the paper's clarity in response to our other reviewer.

# Peer review: Reviewer response for version 2

**DOI:** 10.21956/wellcomeopenres.16420.r34678

**Published:** 2019-02-11

**Authors:** Livia Puljak

**Affiliations:** 1 Cochrane Croatia, Catholic University of Croatia, Zagreb, Croatia

After reviewing the manuscript ‘The development and evaluation of an online application to assist in the extraction of data from graphs for use in systematic reviews’, here is my opinion: this is a relevant manuscript, as the process of evidence synthesis would benefit from further methodology refinements, and certainly new ideas for making systematic review methods faster and more accurate are very welcome.

 I would like to suggest the following minor revisions:

**Abstract**

  

- The extraction of statistical data from graphs in PDF files is’: here, I would remove ‘in PDF files’ because some manuscripts do not have online edition so one would need to rely on printed version. Also, some older manuscripts that are online do not have a PDF version for download. Now, this can be removed if the tool can accept other files too. But if not, it should be specified that the tool in this version was developed only to work with pdf files.
- Please specify clearly in the abstract what is the ‘comparison to standard practice’. I am not sure that there is a standard practice here in a general sense. Many researchers simply disregard data from figures as a ‘practice’. Even in some Cochrane reviews one can find a note that data presented only as graphs will be excluded.
- Later in the text I found more detailed description about what was considered a ‘standard practice’ and I noticed that four electronic data extraction methods were used, and that ruler-paper method was not used. I think this should be clearly specified in the abstract; that the new workflow was compared with other electronic solutions for data extraction that the participants were using most commonly in their research work.
- It would be great to mention already in the abstract how many participants took part in the evaluation of design. I would also mention in the abstract that you had qualitative survey with participants. These are all relevant information to present your study more comprehensively. 
- Explain what does it mean ‘users’ prior preferred current approaches’ and how does it compare to ‘standard practice’ from the previous paragraph of the introduction. See comment above. These terms in abstract should be clearly specified.
- This statement is very unhelpful ‘there may be a saving in time of around 6 minutes per graph,’ because there are different types of graphs and savings of time will not be the same if one graph has ten data points to extract, or twenty, or only three, for example. It would be better to be very clear about where is the time savings – how much time is saved to extract one data point if the authors really want to present time saved. This information about six minutes per graph needs to be removed and revised. For example, you can talk about savings per one data point, or to simply say in the abstract that time was shorter with your tool, but refrain from this kinds of inaccurate statements.

 

**Introduction**

- Do you have a reference for this sentence: ‘This can make the process very time consuming, even for small reviews; the labour required is obviously compounded in very large reviews, such as those seen in preclinical research.’
- Information provided later on, about 6000 studies for a corresponding research could be provided here in the Introduction, i.e. moved from its current position in the manuscript. 
-It would be beneficial to explain in the introduction what is the motive for this work when free and easy-to-use tools such as Plot Digitizer exist? 
- Please elaborate further would be a motivation to make ‘a tool which is designed specifically for data extraction in the context of systematic reviews’. It could be argued that it is irrelevant what is the purpose of data extraction [perhaps to enable some functionalities that the desktop software does not have?]. Perhaps, also to mention methodological research, i.e. research on research – there are potentially more types of studies that would need data from figures.

 

**Research questions**

- Please identify who are ‘participants’ here.

**Identifying graph types**

- ‘we selected 34 papers identified in the context of systematic’: it would be good to provide the list of those papers in the supplementary file

**Design**

- ‘would be extracting data from a PDF file’: I would remove emphasis on pdf files. Or clearly specify that this tool is only for figures in PDF files. 

**Evaluation design**

- I would suggest to explain already here how many participants you had in this evaluation exercise

 

**Discussion**

- Sentence: ‘Our study was not designed to show superiority, but suggests that there may be an average saving in time of around 6 minutes per graph’: the same comment for this time as in the abstract. I would only write about time savings but not provide here minutes per graph. Not every graph will be the same, and then the savings will not be the same for each one.
- Can you explain why is the new tool more accurate than the electronic tools that the participants used as their current method? What is it in those other electronic methods that is inferior, compared to the new tool?
- Kadic et al., should be corrected to Jelicic Kadic et al.
- Section where the manuscript of Jelicic Kadic et al. is mentioned should be revised. The Jelicic Kadic et al. manuscript describes a study where electronic data extraction tool was compared to a paper-based method. However, here paper-based method was excluded from the comparator methods. Therefore, in this study the authors have compared a new electronic tool with existing electronic tools.
- I would revise ‘graphical data extraction tool to current methods of data extraction’ into: ‘new data extraction tool, described hereby, to several desktop electronic methods of data extraction that are currently in use’. 
- It would be nice if the authors could come up with a name for this new tool, so that we do not have to refer to it as a ‘new graphical data extraction tool’.
- I would suggest revising all these ‘nearly 6 minutes in time’ from the Discussion, to simply talk about less time needed with the new tool.

 

**Future work**

- Integration with RevMan would also be nice.

 

 Nice work, overall. Congratulations to the authors.

I have read this submission. I believe that I have an appropriate level of expertise to confirm that it is of an acceptable scientific standard.

# Response

**Published:** 2019-03-02

**Authors:** James Thomas

**Affiliations:** University College London, UK

We would like to thank Livia Puljak for her helpful review and suggestions for improving the paper. We have made the following changes.

Abstract.

1. *We agree that PDF is not the only format / medium used, but this is the area where we see most need, and made not change to the abstract. However, we have clarified the scope of our evaluation on page 1: “While we acknowledge that there are a range of different ways that papers are presented (from online to paper only), we focused this work on the challenge of extracting data from papers published using the PDF file format. This format is the most ubiquitous electronic publishing medium for papers and we therefore see greatest efficiencies from improving software support in this area.”*

2.       We have clarified that we’re referring to participants’ standard practice for extracting data from graphs.

3.       We have clarified that ‘standard practice’ is referring only to extraction of data from pdf documents.

4.       We have added the number of participants to the abstract.

5.       We have replaced ‘prior preferred current approaches’ with ‘standard practice’ to be more consistent.

6. *We have revised the final sentence to read: “Our study was not designed to show superiority, but suggests that, on average, participants saved around 6 minutes per graph using the new tool, accompanied by a substantial increase in accuracy.” We don’t think we can go as far as to say how many seconds / minutes were saved per data point, as there are other factors when considering how long data extraction takes.*

Introduction

1.       I’m afraid we don’t have a reference for that sentence – it is based on personal experience and observation of very many other reviews!

2.       We tried moving that text higher up, but it then spoilt the flow of the argument in the section where it was before, so have left it where it was.

3.       “It would be beneficial to explain in the introduction what is the motive for this work when free and easy-to-use tools such as Plot Digitizer exist”. Thank you – that was indeed a missing piece of our argument! We have added a section into the penultimate paragraph of the introduction to address this point. (This addition also addressed the next point.)

Research questions

1.       “Please identify who are ‘participants’ here.”. In the research questions, we already say that they are people *with a pre-existing interest in and knowledge of systematic review. We have now moved the “Participants and Recruitment” section directly under the “Research Questions” section so that the reader has this information earlier on.*

Identifying graph types

1.       ‘we selected 34 papers identified in the context of systematic’: it would be good to provide the list of those papers in the supplementary file. We have added this information in an additional supplementary file.

Design

1.       As clarified above – we have limited the scope of this evaluation to pdf files.

Evaluation design

1.       We think that this information belongs in the results.

Discussion

1.       We have revised in the same way as above.

2.       “Can you explain why is the new tool more accurate than the electronic tools that the participants used as their current method? What is it in those other electronic methods that is inferior, compared to the new tool?” That is a great question – but we can’t unfortunately! We didn’t investigate this question in the evaluation.

3.       We have revised to clarify that the Jelicic Kadic study compared paper-based to electronic media.

4.       We have revised that sentence as suggested.

5.       We agree that not giving the tool a name is a bit awkward in terms of writing the paper. This was a deliberate choice however, as the tool itself is proof of principle, and not something that systematic reviewers are able to use at the moment.

6.       We can only see one other mention of ‘6 minutes’ in the discussion (apart from the one already revised) and think that this formulation is an accurate reflection of the results. Future work RevMan is really a platform for meta-analysis and review publication, and we do not think its development roadmap includes data extraction (Cochrane uses other tools for this), so haven’t suggested that such a tool should be integrated within RevMan.

# Peer review: Reviewer response for version 1

**DOI:** 10.21956/wellcomeopenres.16057.r34418

**Published:** 2018-12-21

**Authors:** Chris Marshall

**Affiliations:** 1 Institute of Health and Society, Newcastle University, Newcastle, UK

Well written paper, but I’m confused as to where the novelty actually lies here. WebPlotDigitizer is an existing and already very useful tool, and it’s not clear to me what the value of this ‘adapted version’ actually is? Is it the ability to view the PDFs with the graphs directly in the tool? Not sure. I think you need to make clearer the differences between the original software and this evolved version. Also, no proper citation to WebPlotDigitizer!

 I’m also not sure as to what is actually being assessed here: is it an evaluation of this particular tool or are you assessing the concept of using a graph extraction tool to save time (or is it both?).  If it’s the former, it would be good to see a more direct comparison with other similar tools in this space.

 Really appreciated all the links to supplementary files, raw data and code etc. Excellent in terms of transparency and reproducibility.

I have read this submission. I believe that I have an appropriate level of expertise to state that I do not consider it to be of an acceptable scientific standard, for reasons outlined above.

# Response

**Published:** 2019-01-14

**Authors:** James Thomas

**Affiliations:** University College London, UK

We would like to thank Chris Marshall for his helpful feedback. We have modified the article and provided detailed commentary and screenshots which show the novelty of our approach in more detail (we agree that we should have included this in the original). We have also provided a more explicit statement of our research objectives.

# Peer review: Reviewer response for version 1

**DOI:** 10.21956/wellcomeopenres.16057.r34420

**Published:** 2018-12-11

**Authors:** Jon Brassey

**Affiliations:** 1 Trip Database Ltd., Newport, UK

An impressive paper with just minor points...:

 1) "Systematic review and meta-analysis are research techniques whereby all available literature on a research question is collated and analysed to give an overview of that field." I'm thinking that it should include something like "...an attempt is made to locate and use all available literature".  

 2) "there is the clear potential to improve both the speed and accuracy of extraction of outcome data from the included studies once the required outcomes have been identified" - I'd say it's not clear if you're not immersed in automated methods.

 3) Cochrane collaboration - hasn't it dropped 'collaboration'? I appreciate you made the 'c' in 'collaboration' lower case!

 4) "...and are used frequently in clinical decision making" - can we have a reference please?

 5) Because of the challenges in manual data extraction from ROC/AUC graphs, these were not offered in this set - I'd have this as a new paragraph.

 6) Exploration of drop-outs. 32 signed consent and ten completed the trial - I'm sure it's for mundane reasons - but might this high-level of drop-out be down to some unforeseen reason that might impact future roll out of the technology?

 7) Possibly not one for the paper but I do think the long-term future is ensuring the trial variables (including data used in plotting graphs) should be embedded, in computer-readable format, in the articles meta-data. That removes the problem.

I have read this submission. I believe that I have an appropriate level of expertise to confirm that it is of an acceptable scientific standard.

# Response

**Published:** 2019-01-14

**Authors:** James Thomas

**Affiliations:** University College London, UK

We would like to thank Jon Brassey for his helpful feedback. We agree with all his comments and have modified the article to integrate them all - with the exception of the last one: we agree, but this requires longer-term changes to publication practice!
